# High risk for life-threatening adverse events of fluoroquinolones in young adults: a large German population-based cohort study

**DOI:** 10.1186/s12916-025-03919-0

**Published:** 2025-02-07

**Authors:** Julia Wicherski, Jonas Peltner, Cornelia Becker, Katrin Schüssel, Gabriela Brückner, Andreas Schlotmann, Helmut Schröder, Winfried V. Kern, Britta Haenisch

**Affiliations:** 1 Federal Institute for Drugs and Medical Devices (BfArM), Pharmacoepidemiology, Kurt-Georg-Kiesinger-Allee 3, Bonn, 53175 Germany; 2 German Centre for Neurodegenerative Diseases (DZNE), Pharmacoepidemiology in Neurodegenerative Disorders, Bonn, 53127 Germany; 3 Research Institute of AOK (WIdO), Berlin, 10178 Germany; 4 Division of Infectious Diseases, Department of Medicine II, Faculty of Medicine and Medical Centre, University of Freiburg, Freiburg, 79106 Germany; 5 Centre for Translational Medicine, University of Bonn, Bonn, 53113 Germany

**Keywords:** Fluoroquinolones, Antibiotics, Adverse drug reactions, Real-world evidence, Cohort study

## Abstract

**Background:**

Fluoroquinolone antibiotics have a high potential for serious adverse drug reactions, but real-world evidence in European patient cohorts is lacking. Therefore, we aim to examine the association between fluoroquinolone exposure and potentially life-threatening adverse events stratified by age and gender in Germany.

**Methods:**

We conducted an administrative cohort study using the active comparator new user design with a risk window up to 365 days between January 2013 and December 2019. Population-based longitudinal data from one of the largest German statutory health insurances were used. Episodes of newly dispensed fluoroquinolones (ciprofloxacin, levofloxacin, ofloxacin, moxifloxacin, norfloxacin, and enoxacin) were compared to other antibiotics (amoxicillin, amoxicillin clavulanic acid, azithromycin, cefuroxime, cephalexin, clindamycin, sulfamethoxazole-trimethoprim, and doxycycline). Endpoints were defined by incident diagnoses of aortic aneurysm/dissection, cardiac arrhythmia, hepatotoxicity, and all-cause mortality. Adjusted hazard ratios were estimated from piece-wise exponential additive mixed models with smooth non-linear effects for person-time and age and adjusted for comorbidities, year and quarter at index.

**Results:**

The cohorts comprised 15,139,840; 11,760,159; 11,027,175; and 15,305,757 antibiotic episodes. Patients during fluoroquinolone episodes were older (59 versus 51 years) and more often female (58% versus 54%). We counted 46,502; 446,727; 19,125; and 474,411 incident endpoints. Relative risk for all-cause mortality and hepatotoxicity was high for < 40-year- and 40–69-year-old females (aHR = 1.77, 95% CI 1.55–2.03 and aHR = 1.42, 95% CI 1.32–1.53), respectively. For aortic aneurysm/dissection a nominally increased relative risk for < 40-year-old females was found (aHR = 1.42, 95% CI 0.96–2.11), although 95% CI indicates that a small relative risk reduction is also supported by the data. Relative risk for cardiac arrhythmia was increased for men aged < 40 years (aHR = 1.14, 95% CI 1.08–1.20). High relative risks for each endpoint were also identified depending on choice of active comparator, and risks increased with higher defined daily doses and shorter follow-up.

**Conclusions:**

This study contributes real-world evidence to endpoint-specific differences of risks in patient subgroups which need to be considered to improve fluoroquinolone drug safety.

**Supplementary Information:**

The online version contains supplementary material available at 10.1186/s12916-025-03919-0.

## Background

The evidence for serious adverse drug reactions of fluoroquinolones has increased in the last decade. Reassessment of the evidence by the Europeans Medicines Agency (EMA) led to changes and restrictions in the authorization of this antibiotic class in 2019 [1]. Fluoroquinolones are highly effective broad-spectrum antibiotic agents and are considered reserve antibiotics [2], but they continue to be prescribed frequently even after the change in authorization [3].

Irrespective of potential mechanisms leading to these adverse drug reactions (i.e., mitochondrial deoxyribonucleic acid (DNA) or parenchymal cell damages, degrading effect on collagen, diluted extracellular matrix, chelate complexes, human ether-a-go-go-related gene (hERG) potassium channel blocking [4–8]), evidence from previous pharmacoepidemiological studies such as referenced in the EMAs risk assessment report left certain gaps in knowledge regarding differences by age, especially young adults, as well as age-gender-interactions, information on all available broad-spectrum antibiotics as potential reference drugs and information based on routine health care in large European countries. Thus, the primary objective of this population-based cohort study was to provide first insights into fluoroquinolone-associated potentially life-threatening events in German routine care. Therefore, we examined the relative risk of fluoroquinolone-associated aortic aneurysms and dissections, cardiac arrhythmias including sudden cardiac death, acute toxic liver injuries including acute liver failure, and all-cause mortality to contribute to real-world evidence of potentially life-threatening outcomes associated with fluoroquinolones. The secondary objective was to explore specific aspects regarding study design and patient characteristics which may modify the associations between fluoroquinolone exposure and the outcomes of interest.

## Methods

### Source of data

This population-based cohort study was carried out with longitudinal routine billing data from one of the largest association of 11 statutory health insurances in Germany, the “*AOK – Die Gesundheitskasse*”, which covers about 28 million individuals [9]. AOK insurance data are a long-established data source for pharmacoepidemiological studies in Germany [10, 11]. Health insurance is compulsory for all individuals registered in Germany. Around 90% of the population are covered by statutory health insurance. The remaining approximately 10% of the German population are covered by private health insurance [12].

The dataset provided for this study covers individuals with an AOK insurance period during 1 January 2013 to 31 December 2019 and comprised information on date of birth, self-reported gender, quarterly outpatient and weekly inpatient medical diagnoses, hospitalizations and ambulatory drug reimbursement data, including the date of dispensing, the *anatomical therapeutic chemical classification* (ATC) code, and the amount of drug quantified by defined daily doses (DDD) (the German adaptation of World Health Organization (WHO)-ATC classification with DDDs) [13]. Medical diagnoses are coded in the German modification of International Statistical Classification of Diseases and Related Health Problems 10th version (ICD-10-GM), and hospital procedures are classified by OPS (the German adaptation of the International Classification of Procedures in Medicine) [13].

### Cohort

**Fig. 1:**
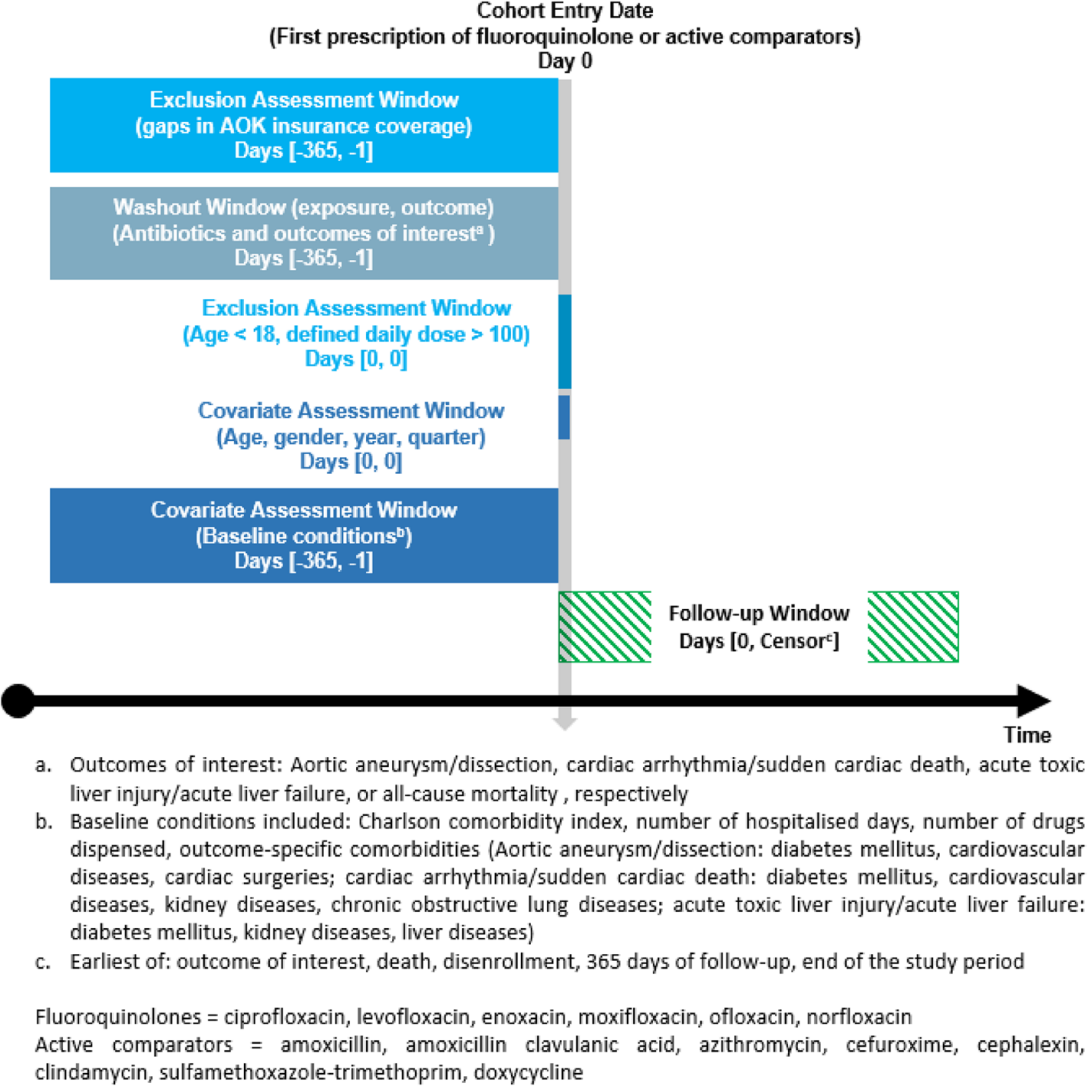
Study design diagram (illustration based on template from Schneeweiss et al. [14])

This cohort study is based on the active comparator new user design. Accordingly, the cohort entry date is defined as the first dispensed prescription of a fluoroquinolone or an active comparator drug (i.e., another broad-spectrum antibiotic agents) (Fig. 1). In order to ensure the inclusion of only new antibiotic users (to avoid prevalent user bias), we identified the first dispensed prescription as index episodes after a 1-year washout period. Individuals had to be continuously covered by the AOK for a baseline period of 365 days and ≥ 18 years of age at their cohort entry date. Patients were excluded if a diagnosis of the outcome of interest was present in the baseline year. Outpatient outcomes which occurred in the same quarter as the index episode were excluded—due to the fact that outpatient diagnoses in the dataset are only available on a quarterly basis, it was not possible to assess whether these outcomes occurred before or after the index antibiotic prescription. Moreover, we defined individuals with an implausible drug amount for antibiotic indications and thus excluded index episodes with more than 100 DDDs dispensed.

All individuals were allocated into four separate outcome-specific cohorts. Multiple inclusion into the cohorts was possible. Individuals were followed-up in a risk window of up to 365 days after cohort entry. The incident occurrence of an outcome of interest was observed in the outcome-specific cohorts. Outcomes of interest were selected from the risk assessment report of the EMA [1]. In scope of this study, we only selected potentially life-threatening outcomes. Even if the incidence of some of these events is rare, public health relevance results from the high number of individuals at risk, i.e., those exposed to fluoroquinolones. ICD-codes used to define the outcomes of interest are shown in Additional file 1: Table S1. We defined cases from outpatient diagnoses with the diagnostic modifier “confirmed” or from inpatient discharge diagnoses. All covariates were assessed during the baseline period. As a general measure of comorbidity, the Charlson comorbidity index (CCI) was determined [15, 16]. In addition, the number of drugs dispensed and hospital days were assessed as further health status-related variables. Outcome-specific comorbidities were defined as comorbidities associated with the outcome of interest. They were assessed based on the presence of diagnoses and/or medical procedures with details described in Additional file 1: Table S1.

### Statistical analysis

Differences in baseline characteristics for all covariates were described by absolute and relative frequencies. Differences between patient characteristics in fluoroquinolone and active comparator episodes were quantified by standardized differences. Good balance was defined as standardized differences ≤ 0.2. Incidence rates were calculated and standardized for age and gender based on the population of the German Census 2011 [17] by applying direct standardization with Poisson approximation [18].

Piece-wise exponential additive mixed models (PAMM) [19] were applied to all outcome-specific datasets. A PAMM models the baseline hazard using a smooth, non-linear function. All models for the different outcomes included age (at baseline) and individual person-time as smooth nonlinear time-constant confounders as well as all other baseline characteristics as linear time-constant confounders.

We conducted pre-planned subgroup analyses to disaggregate our results by gender, age, and gender and age groups combined. Outcomes of interest in the main analyses were defined by inpatient and outpatient diagnoses, of which the latter are available only on a quarterly basis. Therefore, a sensitivity analysis was conducted using only inpatient diagnoses with higher temporal resolution (per week) and allowing for analysis of shorter risk windows (up to 30, 60, 92 days, respectively). To further increase balance of baseline characteristics and investigate exposure misclassification as well as active comparator group and endpoint definition, several pre-planned sensitivity analyses were applied. In order to further increase balance of baseline characteristics between fluoroquinolone and active comparator treatment groups, propensity score matching was applied. Propensity scores were based on a logistic regression model considering all baseline characteristics, and a 1:1 nearest neighbor propensity score (PS) matching with a caliper of 0.1 was applied. Furthermore, for the cohort of all-cause mortality, we additionally excluded all individuals with a CCI ≥ 1, with dispensed drugs or with hospitalization in the baseline year in order to restrict analyses to a population as healthy as possible.

Several sensitivity analyses were conducted to investigate exposure misclassification: in a first analysis, observation times were censored, if a new antibiotic prescription for a different antibiotic drug occurred within the follow-up time (“per protocol censoring”). Furthermore, we excluded all individuals with a hospitalization during the baseline period, in order to reduce possible misclassification by inpatient antibiotic exposure, for which data is unavailable in German administrative health claims data. In addition, patients were split into subgroups according to length of hospital stays during follow-up period (0 days, 1 to 7 days, 8 and more days). Dose effects were approximated by DDD categories (drug-specific: low, medium, high).

Two more types of sensitivity analyses were conducted to investigate the active comparator group and outcome definitions in more detail: (1) we disaggregated our active comparator group into pairwise PAMMs for each active comparator agent (active ingredient) versus fluoroquinolones to check whether the choice of comparator drug matters, and (2) in sensitivity analyses on the outcome definition, more specific subsets of ICD-10-GM codes were used for outcome definition (i.e., aneurysms and dissections separately, different types of cardiac arrhythmia and sudden cardiac death separately, acute toxic liver diseases and acute liver failure separately).

Statistical analyses were conducted in R, version 4.1.0 from February 2022 to April 2024. Propensity score matching was conducted in SAS, version 9.4.

## Results

The four cohorts on aortic aneurysm and dissection (aortic events), cardiac arrhythmia including sudden cardiac death (cardiac events), acute toxic liver diseases including acute liver failure (hepatic events), and all-cause mortality comprised overall 15,139,840; 11,760,159; 11,027,175; and 15,305,757 antibiotic index episodes, which fulfilled each of the applied selection criteria. Among these cohorts, the proportion of fluoroquinolone episodes ranged from 22 to 24%, as illustrated in Additional file 1: Fig. S1. Baseline characteristics for the four cohorts are displayed in Additional file 1: Tables S2a-b. Fluoroquinolone and active comparator groups were well balanced for gender (standardized differences ≤ 0.2). Male patients comprised 42% of all fluoroquinolone episodes and 46% of all active comparator episodes. Meaningful differences were observed for age (standardized differences > 0.2) across all four datasets. The mean age of patients with fluoroquinolone episodes (56–59 years) was higher compared to active comparator episodes (49–51 years). Differences in health status-related variables (i.e., CCI, drugs dispensed and hospitalized days) and outcome-specific comorbidities (i.e., cardiovascular diseases, diabetes mellitus, cardiac surgery, chronic obstructive pulmonary disease, renal and liver diseases) were small with a consistent trend towards higher values indicative of higher frailty or morbidity in patients with fluoroquinolone episodes. Across all study years, proportionally more antibiotics (fluoroquinolones and active comparators) were prescribed and dispensed in winter than in summer months. Moreover, there were small differences in the distribution of fluoroquinolone and active comparator episodes across the cohort entry date years 2014–2018, indicating a declining trend in fluoroquinolones prescribing. Fluoroquinolone episodes in the year 2014 contributed to 22–23% to the total of fluoroquinolone episodes, while the year 2018 comprised only 17%.

### Primary outcome

During the 365-day risk window, 46,502 incident aortic diagnoses of interest, 446,727 incident cardiac diagnoses of interest (of which 3.07% were cardiac arrest), 19,125 incident hepatic diagnoses of interest (of which 51.03% were acute liver failure), and 474,411 deaths were registered in the respective cohorts. The crude incidence rates (IRs) of all four outcomes were higher for fluoroquinolone episodes compared to active comparator episodes, with 40 versus 26 incident aortic diagnoses, 470 versus 325 incident cardiac diagnoses, 24 versus 13 incident hepatic diagnoses and 582 versus 260 deaths, respectively, per 10,000 episodes. After standardization for age and gender, IRs per 10,000 episodes of fluoroquinolones versus active comparators decreased to 29 (95% CI 26;32) versus 27 (26;29) incident aortic diagnoses, 373 (360;386) versus 349 (341;356) incident cardiac diagnoses, 20 (20;22) versus 13 (12;14) incident hepatic diagnoses, and 324 (314;334) versus 220 (215;225) deaths, respectively. The standardized IRs indicate small differences between fluoroquinolones and active comparator episodes for aortic and cardiac events, but IRs for hepatic events and all-cause mortality remained higher in fluoroquinolone episodes compared to active comparator episodes after standardization.

Table 1 shows the results of the main PAMMs. Fluoroquinolone episodes had slightly increased risks for experiencing an outcome in all models. Male gender was a risk factor for all four outcomes of interest; especially for the occurrence of aortic events the adjusted hazard ratio (aHR) was threefold higher compared to females. Risk of hepatic events and all-cause mortality increased with higher CCI scores. There was a positive trend regarding the total number of drugs dispensed during the baseline year: the more drugs an individual was exposed to, the higher the risk of an adverse outcome.

**Table 1:** Main results from PAMM regression models

	**Cohort 1:**		**Cohort 2:**		**Cohort 3:**						**Cohort 4:**		
	**Aortic aneurysm/dissection**		**Cardiac arrhythmia/sudden cardiac death**		**Acute toxic liver injury/acute liver failure**						**All-cause mortality**		
	**aHR**	**[95% CI]**	**aHR**	**[95% CI]**	**aHR**	**[95% CI]**					**aHR**		**[95% CI]**
Fluoroquinolone episode (ref. active comparators)	1.07	[1.04;1.09]	1.05	[1.04;1.05]	1.34		[1.30;1.39]				1.23	[1.23;1.24]	
Males (ref. females)	3.03	[2.96;3.10]	1.23	[1.22;1.24]	1.38		[1.33;1.42]				1.47	[1.46;1.48]	
CCI (ref. 0)													
1–2	1.11	[1.09;1.14]	0.97	[0.96;0.98]	1.10		[1.06;1.14]				1.72	[1.71;1.74]	
3–4	1.10	[1.07;1.14]	0.96	[0.94;0.97]	1.16		[1.09;1.24]				2.33	[2.30;2.35]	
5 +	1.01	[0.97;1.06]	1.02	[1.00;1.04]	2.91		[2.75;3.09]				4.49	[4.44;4.54]	
Drugs dispensed (ref. 0)													
1–3	1.14	[1.07;1.22]	1.19	[1.18;1.21]	1.06		[0.98;1.16]				0.83	[0.80;0.85]	
4–10	1.32	[1.24;1.40]	1.46	[1.44;1.49]	1.28		[1.18;1.38]				1.01	[0.99;1.04]	
11–20	1.46	[1.37;1.54]	1.68	[1.66;1.71]	1.57		[1.45;1.71]				1.32	[1.29;1.35]	
21 +	1.51	[1.42;1.61]	2.14	[2.10;2.17]	2.19		[2.02;2.38]				2.19	[2.14;2.25]	
Hospitalized days (ref. 0)													
1–7	1.10	[1.07;1.13]	1.03	[1.02;1.04]	1.47		[1.40;1.54]				1.33	[1.32;1.34]	
8 +	1.06	[1.03;1.09]	1.11	[1.10;1.12]	2.71		[2.61;2.83]				2.48	[2.46;2.49]	
Cohort entry year (ref. 2014)													
2015	1.02	[0.99;1.06]	0.99	[0.98;1.00]	1.06		[1.01;1.12]				0.96	[0.95;0.97]	
2016	1.06	[1.02;1.10]	0.99	[0.98;1.00]	1.03		[0.98;1.08]				0.97	[0.96;0.98]	
2017	1.13	[1.09;1.17]	0.95	[0.94;0.96]	1.04		[0.99;1.10]				0.96	[0.95;0.97]	
2018	1.27	[1.22;1.31]	0.94	[0.93;0.95]	1.03		[0.98;1.08]				0.95	[0.94;0.95]	
Cohort entry quarter (ref. Q1 (January–March)													
Q2 (April–June)	1.02	[0.99;1.05]	1.03	[1.02;1.03]	1.07		[1.02;1.11]				1.03	[1.03;1.04]	
Q3 (July–September)	1.06	[1.03;1.09]	1.02	[1.01;1.03]	1.04		[1.00;1.09]				1.09	[1.08;1.10]	
Q4 (October–December)	1.07	[1.04;1.10]	1.03	[1.02;1.04]	1.06		[1.02;1.11]				1.05	[1.04;1.05]	
Outcome-specific additional covariables													
Diabetes mellitus	0.74	[0.72;0.76]	1.03	[1.02;1.03]	1.04			[1.00;1.08]			*NA*		
CVD	1.93	[1.88;1.98]	1.44	[1.43;1.45]	*NA*						*NA*		
Cardiac surgery	1.02	[0.93;1.10]	*NA*		*NA*						*NA*		
COPD	*NA*		1.14	[1.13;1.15]	*NA*						*NA*		
Renal diseases	*NA*		1.14	[1.13;1.15]	2.48				[2.40;2.57]		*NA*		
Liver diseases	*NA*		*NA*		1.25				[1.20;1.30]		*NA*		
Approximate significance of smooth terms:													
	edf	*p*-value	edf	*p*-value	edf					*p*-value	edf		*p*-value
Follow-up	2.96	< 0.001	2.91	< 0.001	2.98					< 0.001	3.00		< 0.001
Age in years	6.42	< 0.001	8.27	< 0.001	4.75					< 0.001	8.86		< 0.001

Adjusted hazard ratio (aHR) | 95% confidence interval (CI) [lower confidence level; upper confidence level] | reference (ref.) | Charlson comorbidity index (CCI) | Quarter 1–4 (Q1–4): January–March, April–June, July–September, October–December | cardiovascular disease (CVD) | chronic obstructive pulmonary disease (COPD) | Not applicable (NA) | estimated degrees of freedom (edf)

For cohort entry calendar years, risk for aortic events increased between 2014 and 2018, but this effect did not remain after restriction to inpatient cases in sensitivity analyses. In terms of outcome-specific comorbidities, risk of aortic events was reduced in individuals with diabetes mellitus. All other outcome-specific comorbidities were associated with increased risks for an adverse event of interest, although increases in risk were only small in some cases. Age and an individuals’ person-time were relevant smoothing terms for all four PAMM regressions as displayed at the bottom of Table 1 (and as plots in Additional file 1: Fig. S2). This is indicative of a non-linear relation between those variables and the outcome.

### Subgroup and sensitivity analyses

**Fig. 2:**
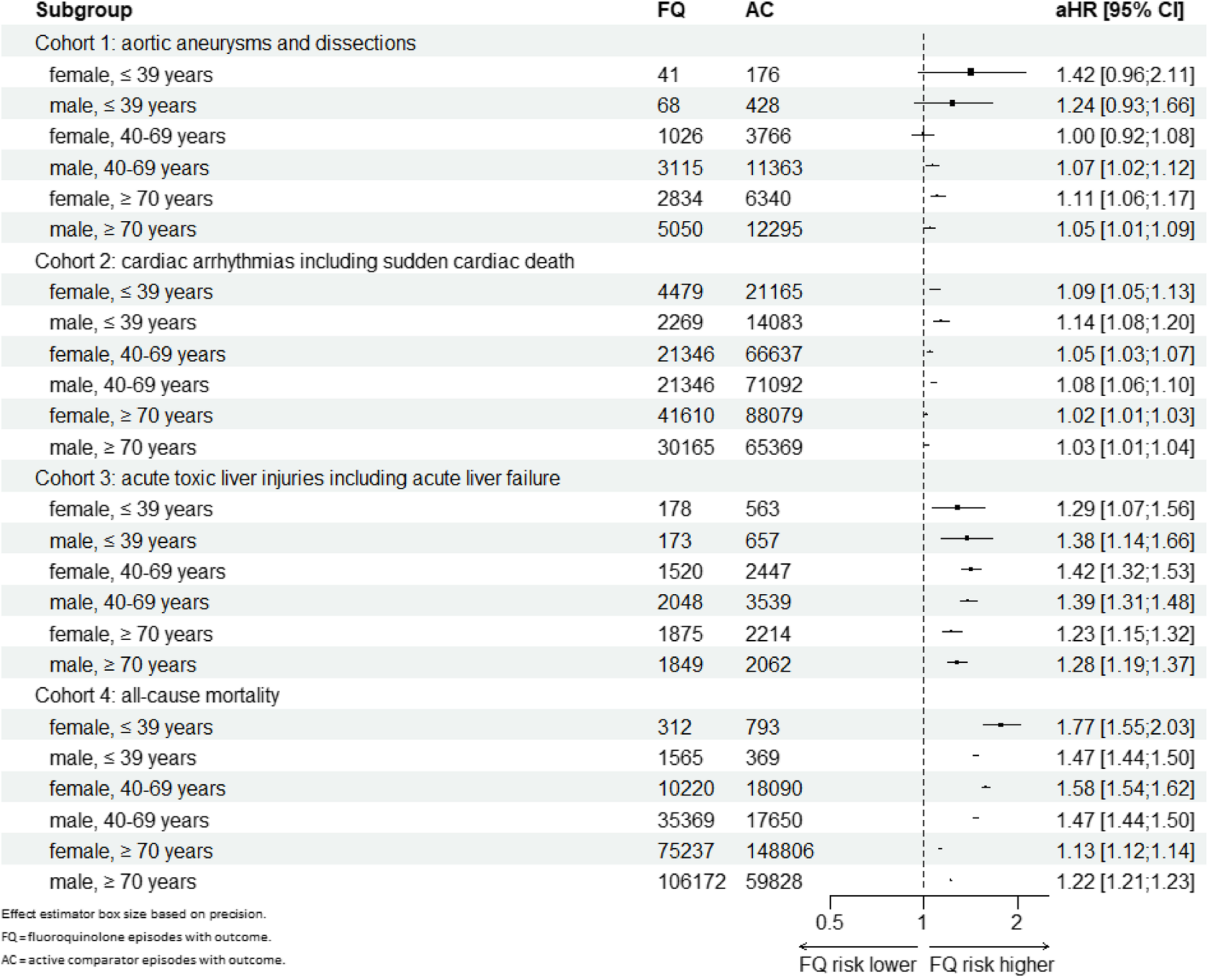
Results from PAMM regression models stratified by age and gender

The subgroup analyses stratified by age and gender (Fig. 2) depict differences in all outcomes of interest, with increased rates seen in fluoroquinolone episodes compared to active comparator episodes, in all age groups except for aortic events in patients < 40 years and females aged 40–69 years. Especially, < 40-year-old female individuals with a fluoroquinolone episode had an increased risk of all-cause mortality within 365 days compared to those with an active comparator episode. The risk for fluoroquinolone-associated hepatic events was highest in 40–69-year-old females. Individuals < 40 years were also at high risk for fluoroquinolone-associated aortic events, although the 95% confidence intervals (CIs) indicate a higher degree of uncertainty of the estimate. Referring to the absolute risk, standardized incidence rates increase with age, but relative risk of fluoroquinolones is higher in 18–39 years old individuals compared to 40–69 and ≥ 70-year-olds for aortic and cardiac events as well as mortality (Additional file 1: Table S3).

In order to analyze the effect of methodological specifications, we restricted our analyses to inpatient adverse events and shortened the follow-up period, in which outcomes were allowed to occur (Table 2). Depending on the outcome of interest, effects varied by the length of follow-up. For all outcomes, except all-cause mortality, the aHRs were highest in the months following the index date and decreased over time. For all-cause mortality, aHRs remained relatively stable.

**Table 2:** Results from PAMM regression models for inpatient outcomes only, stratified by length of risk window

Outcome of interest	aHR [95% CI]			
	**Risk window (follow-up period)**			
	** ≤ *30 days***	** ≤ *60 days***	** ≤ *92 days***	** ≤ *365 days***
Aortic events	1.40 [1.27;1.55]	1.32 [1.22;1.43]	1.30 [1.21;1.39]	1.17 [1.12;1.22]
Cardiac events	1.14 [1.11;1.17]	1.14 [1.11;1.16]	1.13 [1.11;1.15]	1.09 [1.08;1.10]
Hepatic events	1.69 [1.54;1.86]	1.67 [1.55;1.80]	1.61 [1.51;1.72]	1.41 [1.35;1.46]
All-cause mortality	1.24 [1.22;1.26]	1.26 [1.25;1.28]	1.27 [1.26;1.28]	1.23 [1.23;1.24]

adjusted hazard ratio (aHR) [95% confidence interval (CI) [lower confidence level; upper confidence level] | aortic events = aortic aneurysm/-dissection | cardiac events = cardiac arrhythmia/sudden cardiac death |hepatic events = acute toxic liver injury/acute liver failure

Results of PS-matched analyses yielded comparable fluoroquinolone-associated aHRs for all outcomes of interest (see Additional file 1: Tables S4-5). Results of further sensitivity analyses are displayed in Additional file 1: Table S6. For all-cause mortality, aHR for fluoroquinolone-associated death remained elevated in both risk windows even after restricting the analyses to only the healthiest individuals (as a proxy for not being able to fully adjust for comorbidities). The aHRs estimated in our “per protocol” censored analysis, in subgroups of patients with hospitalization during follow-up, and in sensitivity analyses excluding patients with hospitalizations during the baseline year were comparable to the main analysis all outcomes. Analyses stratified by dosage indicated a dose–effect relationship for all outcomes of interest; the largest difference between aHRs by DDD category was observed for hepatic events.

Furthermore, the aHR of fluoroquinolone-associated adverse events may be affected by the choice of comparator drugs (Table 3). During the 365-day risk window, there were only minor differences in the individual contrast for aortic and cardiac events. For hepatic events and all-cause mortality, aHRs were especially elevated when compared with amoxicillin, azithromycin, doxycycline, and clindamycin. Restricting the risk window to 92 days with outcomes based on inpatient diagnoses, fluoroquinolone-associated risks for aortic and cardiac events were elevated relative to clindamycin, doxycycline, azithromycin, and amoxicillin. The risk for hepatic events was increased for fluoroquinolone episodes compared to most comparator drugs. For all-cause mortality, patients during fluoroquinolone episodes were more likely to die compared to all comparator drugs except for amoxicillin-clavulanic acid.

**Table 3:** Results from PAMM regression models stratified by single active comparator drug agents, cohorts 1–4

Active comparator agent	Cohort 1: aortic aneurysm/dissection		Cohort 2: cardiac arrhythmia/sudden cardiac death		Cohort 3: acute toxic liver injury/acute liver failure		Cohort 4: all-cause mortality	
	**aHR**	**[95% CI]**	**aHR**	**[95% CI]**	**aHR**	**[95% CI]**	**aHR**	**[95% CI]**
***365-day risk window***^a^								
Amoxicillin	1.02	[0.98;1.05]	1.07	[1.06;1.08]	1.41	[1.35;1.48]	1.37	[1.36;1.38]
Amoxicillin-clavulanic acid	0.97	[0.93;1.01]	0.96	[0.95;0.97]	1.06	[1.01;1.12]	0.93	[0.92;0.94]
Azithromycin	1.11	[1.06;1.17]	1.08	[1.07;1.10]	1.26	[1.22;1.31]	1.60	[1.58;1.63]
Cephalexin	0.99	[0.80;1.24]	1.08	[1.00;1.16]	1.04	[0.80;1.35]	1.49	[1.39;1.60]
Cefuroxime	1.04	[1.01;1.08]	0.99	[0.98;1.00]	1.19	[1.14;1.24]	1.11	[1.10;1.12]
Clindamycin	1.18	[1.12;1.23]	1.12	[1.11;1.14]	1.46	[1.38;1.55]	1.59	[1.57;1.61]
Sulfamethoxazole-trimethoprim	1.06	[1.01;1.12]	1.04	[1.02;1.05]	1.10	[1.04;1.17]	1.01	[1.00;1.02]
Doxycycline	1.14	[1.09;1.19]	1.09	[1.07;1.10]	1.60	[1.51;1.69]	1.57	[1.55;1.59]
***92-day risk window***^b^								
Amoxicillin	1.36	[1.23;1.51]	1.21	[1.18;1.24]	1.96	[1.78;2.16]	1.38	[1.35;1.40]
Amoxicillin-clavulanic acid	0.93	[0.83;1.04]	0.83	[0.81;0.86]	1.06	[0.97;1.17]	0.87	[0.86;0.89]
Azithromycin	1.60	[1.38;1.86]	1.41	[1.36;1.47]	1.78	[1.53;2.08]	1.72	[1.67;1.76]
Cephalexin	1.76	[0.78;4.00]	1.29	[1.07;1.56]	1.03	[0.63;1.68]	1.72	[1.52;1.96]
Cefuroxime	1.16	[1.06;1.27]	0.99	[0.96;1.01]	1.41	[1.30;1.53]	1.09	[1.08;1.11]
Clindamycin	1.57	[1.36;1.80]	1.43	[1.38;1.48]	2.37	[2.08;2.71]	1.94	[1.89;1.99]
Sulfamethoxazole-trimethoprim	1.20	[1.03;1.39]	1.08	[1.04;1.12]	1.10	[0.99;1.23]	1.07	[1.05;1.09]
Doxycycline	1.71	[1.48;1.96]	1.40	[1.35;1.45]	2.40	[2.10;2.75]	1.71	[1.67;1.75]

adjusted hazard ratio (aHR) | 95% confidence interval (CI) [lower confidence level; upper confidence level]

^a^Main model, including outpatient and inpatient diagnoses

^b^Sensitivity, including inpatient diagnoses for outcomes 1 to 3 only

In a final sensitivity analysis, outcome diagnoses were disaggregated to smaller diagnostic groups (Table 4). Within the aortic diagnosis groups, the aHR for aneurysms remained the same while for dissections the association for fluoroquinolone episodes compared to active comparator episodes increased. With regard to cardiac diagnoses, the aHRs were increased for fluoroquinolone-associated cardiac arrest and paroxysmal tachycardia. For fluoroquinolone-associated liver damage, the aHR was higher for acute liver failure than for other hepatic events.

**Table 4:** Results from PAMM regression models stratified by outcome diagnoses, cohorts 1–3

Cohort	Diagnosis	aHR	[95% CI]
1	Aortic aneurysm	1.06	[1.04;1.09]
	Aortic dissection	1.15	[1.04;1.29]
2	Atrioventricular and left bundle-branch block	0.98	[0.96;1.01]
	Other conduction disorders	1.03	[1.00;1.06]
	Cardiac arrest	1.12	[1.07;1.16]
	Paroxysmal tachycardia	1.10	[1.08;1.13]
	Atrial fibrillation and flutter	1.06	[1.04;1.07]
	Other cardiac arrhythmias	1.04	[1.02;1.05]
3	Acute liver failure	1.44	[1.37;1.50]
	Other acute toxic liver diseases/injuries	1.24	[1.18;1.30]

adjusted hazard ratio (aHR) | 95% confidence interval (CI) [lower confidence level; upper confidence level]

## Discussion

To the best of our knowledge, this is the first cohort study on fluoroquinolone-associated potentially life-threatening adverse events in an active comparator new user design in a large central European country. Due to the large dataset, detailed analysis with regard to the choice of antibiotic comparator drug as well as subgroup-specific risks by age and gender could be performed. After covariate adjustment and propensity score matching, elevated risks for fluoroquinolones for all analyzed adverse events were found. Younger individuals (18–39 years old) with fluoroquinolone episodes were found to be at high relative risk for associated outcomes of interest, but the absolute risk of the outcome occurrence is increasing with age. Stratified by single active comparator drugs, we found differences in the strength of the association between fluoroquinolones and reference drugs for each of the four outcomes analyzed. With our findings in a German cohort, we add information to the literature regarding fluoroquinolone risks in a large central European country and provide first insights into risks in younger adults (< 40 years old) for the most of our outcomes of interest.

Previous active comparator new user studies on fluoroquinolone-related adverse events [20–25] assessed single active comparator agents and applied different study population selection criteria. Fluoroquinolone-associated aortic aneurysm and dissection were compared to amoxicillin in a Swedish cohort [24], and in different United States (US) populations compared to macrolides [20], to azithromycin, combined trimethoprim and sulfamethoxazole, and amoxicillin [21], and to a group of active comparator agents [26]. Compared to Pasternak et al. [24], who reported an increased relative risk of 1.66 [1.12;2.46] for fluoroquinolones compared to amoxicillin during 60 days of follow-up, the relative risk is lower in Germany based on our result of the sensitivity analysis regarding single-agent comparison during 92 days of follow-up (aHR = 1.36 [1.23;1.51]). Moreover, the risk in Germany is lower in comparison to azithromycin and amoxicillin but higher in comparison to trimethoprim-sulfamethoxazole combination reported in US population [21]. Gopalakrishnan et al. [21] reported an aHR of 2.57 [1.36;4.86] compared to azithromycin, 1.54 [1.33;1.79] compared to amoxicillin, and 0.99 [0.62;1.57] compared to trimethoprim-sulfamethoxazole combination during 60 days of follow-up, whereas respective aHRs for Germany were 1.60 [1.38;1.86], 1.36 [1.23;1.51], and 1.20 [1.03;1.39] during 92 days of follow-up. Additionally, Garg et al. [20] used all macrolides as active comparators to fluoroquinolones and estimated an aHR of 1.34 [1.17;1.54] for aortic aneurysm and dissection events during 60 days of follow-up. This effect estimate agrees with the effect estimate derived by our German 60-day risk window analysis with different active comparative agents (aHR = 1.32 [1.22;1.43]), but relative risk is higher in Germany if we only reference to the single-agent comparison to azithromycin (60% increased relative risk during 92-day follow-up). One explanation for higher risks observed in our German data is the fact, that not only ≥ 50 years old individuals were included in the analysis as it was the situation in studies provided by Pasternak et al. [24] and Gopalakrishnan et al. [21]. The latter study, furthermore, selected only pneumonia and urinary tract infections for azithromycin and trimethoprim-sulfamethoxazole combination comparison, respectively [21]. Azithromycin is just one macrolide, the increased risk in German comparison to azithromycin compared to all macrolides used by Garg et al. [20]. In terms of study design, our results are most comparable to the US population studied by Newton et al. [26]. However, unlike them, our current study also covered individuals aged 65 years and older, and we additionally included doxycycline and amoxicillin as comparator drugs and stratified our analysis for all single active agent comparisons. Despite these differences in the reference group selection and the age distribution of participants, results of our 92-day risk window (aHR = 1.30 [1.21;1.39]) are comparable to Newton et al.’s (aHR = 1.20 [95% CI 1.17;1.24]). However, our inconclusive but increased effect estimate regarding high relative risk in young individuals (i.e., 18–39 years old) is not supported by Newton et al. [26]. They found no association for 18–34 years old individuals but increased relative risk for 35–49-year-olds analyzed with a larger number of episodes at risk and indication included as covariate [27]. The age group (18–39 years) was not analyzed by other previous studies. For aortic dissections, estimates of relative risks are also divergent: while previous studies [21, 22, 25, 27] found no association for dissections only, our German data displayed a 15% increased relative risk for aortic dissection only. Moreover, Aspinall et al. [27] reported increased relative risks for fluoroquinolone-associated aortic aneurysms and mortality, but no difference in the likelihood of ventricular arrhythmia in a US population based on a self-controlled case series analysis compared to different single active comparators. In scope of cardiac arrhythmia, the Danish-Swedish bi-national cohort study by Inghammar et al. [22] also found no association between fluoroquinolones and ventricular arrhythmia at any time point compared to penicillin V. However, a Taiwanese population-based study reported increased risks for ventricular arrhythmia or cardiovascular death for moxifloxacin (aOR = 2.23 [1.71;2.90]) but not for levofloxacin (aOR = 1.20 [0.96;1.49]) and ciprofloxacin (aOR = 0.75 [0.59;0.94]) compared to amoxicillin-clavulanic acid during 30 days of follow-up. Relative risks were higher in shorter follow-up windows of 7 and 14 days [28]. Another study, based on US veteran population (88% males), also reported an increased relative risk for cardiac arrhythmia for levofloxacin compared to amoxicillin during short follow-up windows of up to 10 days (aHR_1–5 days_ = 2.43 [1.56; 3.79] and aHR_6–10 days_ = 1.75 [1.09; 2.82]) but not azithromycin (aHR_1–5 days_ = 1.37 [0.88;2.13] and aHR_6–10 days_ = 1.28 [0.79;2.08]) [29]. Compared to our German findings, relative risks for fluoroquinolones versus amoxicillin were smaller (aHR = 1.21 [1.18;1.24]), but we estimated an increased risk compared to azithromycin (aHR = 1.41 [1.36;1.47]) during 92 days of follow-up. It could be assumed that the risk window of fluoroquinolone-associated arrhythmic events is highest the first days after exposure. We were able to describe this association with a larger sample size and observed that especially the relative risk for paroxysmal tachycardia as well as cardiac arrest is increased in fluoroquinolone episodes compared to a group of active comparators and compared to single active ingredients. Overall, the results of the individual comparisons of the active comparator agents of all four outcomes of interest indicate that there is an impact of the individual drug agent itself or there is a non-random treatment decision between antibiotic agents from one substance class such that the leading condition might have an impact on the likelihood of the outcomes (confounding by indication), which should be considered. Acute liver injuries were associated with fluoroquinolone exposure compared to amoxicillin in Swedish cohort study and compared to clarithromycin in a Canadian study [23, 25], but these studies were restricted to certain age groups and were based on comparatively small numbers of events. The cohort by Nibell et al. [24] reported an aHR of 2.32 [1.01;5.35] for fluoroquinolones compared to amoxicillin during 60 days of follow-up in 40–85 years old individuals in Sweden, but their analysis was based on only 26 acute liver injury events. Findings by Paterson et al. [26] in a nested case–control study of ≥ 66-year-olds with 144 acute liver injury cases show also increased odds ratios of 2.20 [1.21;3.98] for moxifloxacin, 1.85 [1.01;3.39] for levofloxacin but a non-significant effect for ciprofloxacin (aOR = 1.56 [0.95;2.58]) compared to clarithromycin during 30 days of follow-up. In summary, these studies found an increased risk of liver injury after fluoroquinolone exposure compared to amoxicillin or clarithromycin in 60- or 30-day risk windows. These results are in line with our sensitivity analyses of single agent comparisons during 92 days of follow-up with an aHR = 1.96 [1.78;2.16] compared to amoxicillin and aHR = 1.78 [1.53;2.08] compared to azithromycin (a macrolide antibiotic as clarithromycin). We additionally contribute information on age and gender differences with a large number of adverse events detected and that this association is longer present than 60 days after exposure. To the best of our knowledge, this is one of the first studies analyzing all-cause mortality in an active comparator new user design and on a population-based level. The US veterans study (88% males) by Rao et al. [29] estimated an increased risk for all-cause mortality for levofloxacin compared to azithromycin and amoxicillin during up to 5 and up to 10 days of follow-up with relative risk increases of 71–149%. One further study, again of predominantly male veterans [27], analyzed fluoroquinolone-associated all-cause mortality in a self-controlled case series design and also reported an increased mortality risk for fluoroquinolones compared to amoxicillin (aHR = 1.23 [95% CI 1.16;1.31]), azithromycin (aHR = 1.99 [1.83;2.16]), cefuroxime/cephalexin (aHR = 1.29 [1.19;1.41]), doxycycline (aHR = 1.17 [1.08;1.28]), and sulfamethoxazole-trimethoprim combination (aHR = 1.34 [1.23,1.45]) [27]. These findings are in line with results from our German population-based cohort (aHR_amoxicillin_ = 1.38 [1.35;1.40], aHR_azithromycin_ = 1.72 [1.67;1.76], aHR_cephalexin_ = 1.72 [1.52;1.96], and aHR_doxycycline_ = 1.71 [1.67;1.75]), except for the sulfamethoxazole-trimethoprim combination (aHR = 1.07 [1.05;1.09]). This active ingredient is commonly prescribed in females in Germany, and therefore, indications for male veterans might differ meaningfully. Another difference compared to the study by Aspinall et al. [27] is the large size of our study population and younger mean age. Due to this, our study adds relevant information that young females (< 40 years old) are at high relative risk regarding all-cause mortality after fluoroquinolone exposure, but further studies are needed to support this association.

There are, however, limitations that have to be considered when interpreting the results. Due to the use of a relatively long 365-day risk window, bias towards null due to the duration of lagged antibiotic exposure effects may occur if adverse drug effects develop during a short time-interval after exposure. Additionally, information on drug dispensing was used as a proxy of exposure, but non-adherence may be present in some cases, leading to exposure misclassification, which again can result in bias towards the null which as addressed by our sensitivity analysis on shortened risk windows. German statutory health insurance data provide outpatient diagnoses only on a quarterly basis, leading to interval-censored outcomes and introducing uncertainty to the individual person-time in the regression analyses. However, sensitivity analyses based on inpatient diagnoses with higher temporal accuracy (per week) resulted in comparable effect estimates. Additionally, the PAMM regression is an adequate model to apply to such data, as it allows estimating the hazard of an event using a discretized time variable. German statutory health insurance data does not contain information on the indication for an antibiotic prescription. Thus, a direct linkage between infectious diseases with a corresponding antibiotic prescription is not possible. As an infectious disease itself may be a risk factor for development of the outcomes of interest, confounding by indication has to be considered. Especially in the context of all-cause mortality, it needs to be taken into account that indications triggering a fluoroquinolone prescription reflect more likely individuals with severe infections (e.g., infections with multidrug-resistant bacteria) and, therefore, those individuals might have a higher baseline risk for all-cause mortality. Furthermore, a screening bias may be present in cases where the study drug indication requires follow-up health examinations such as imaging of the thorax in terms of pneumonia, which may lead to an increased detection rate of aortic aneurysms. Therefore, single active comparator agent analyses of several commonly used broad-spectrum antibiotics was applied to detect differences. Another source of screening bias within the scope of aortic events was the new guideline for abdominal aortic aneurysm ultrasound screening in ambulatory care which was published by the end of 2016 in Germany [30], but we were able to address this with a sensitivity analysis regarding inpatient diagnoses only and additionally including the year of cohort entry in multivariable analyses. Administrative health claims data lack information on important life style factors and health-related behavior such as smoking or very low or high body weight. These factors may affect both pharmacokinetics and may modify risks for adverse events. Fluoroquinolones were only studied as one single drug class, although single active ingredients and especially moxifloxacin may have differential risks than other fluoroquinolones. But since 65% of all fluoroquinolone episodes represented ciprofloxacin and an additional 20% levofloxacin, class effects were assumed, and no fluoroquinolone-stratified analysis was planned. By using an active comparator new user design, we have reduced the risk of confounding by indication, since comparable antibiotics were used as reference group. The new user design minimizes selection effects such as accumulation of patients tolerating a drug that may be present in prevalent users. With adjustment for several covariates and propensity score matching, we took further steps to appropriately minimize confounding by baseline conditions and the problem of non-random treatment allocation in observational studies. A further strength of the present study is the large dataset allowing for detailed analyses. Moreover, healthcare access within the statutory health system in Germany is not limited by, e.g., financial constraints. We therefore expect a very high degree of certainty that all relevant cases will have been recorded.

## Conclusions

In summary, fluoroquinolone episodes are associated with increased risks for all outcomes. Even if some of these outcomes are rare events, these relative risks are clinically relevant since fluoroquinolones are still commonly prescribed and, therefore, the population at risk is very large. Our study contributes insights into fluoroquinolone therapy safety regarding relevant aspects such as age, gender, and alternative antibiotics. The results demonstrate the need of post-marketing surveillance to complement and support potential decision-making processes to improve safety of drug therapy. Further research should elucidate changes in prescribing behavior and associated patient characteristics after restrictions of fluoroquinolones authorization in 2019.

## Supplementary Information

Additional File 1. Table S1. Definition of study variables. Figure S1. Cohort attrition. Table S2a. Study population characteristics, cohort 1-2. Table S2b. Study population characteristics, cohort 3-4. Figure S2. Smooth plots from PAMM regression models, cohort 1-4. Table S3. Age- and gender-standardized incidence rate (per 10,000 episodes), stratified by age groups, cohort 1-4. Table S4a. Study population characteristics, 1:1 PS matched cohort 1-2. Table S4b. Study population characteristics, 1:1 PS matched cohort 3-4. Table S5. Results from PAMM regression models, PS-matched cohorts. Table S6. Results from PAMM regression models in sensitivity analyses, cohort 1-4

## Data Availability

No datasets were generated or analysed during the current study.

## Abbreviations

aHR: Adjusted hazard ratio
AOK: German statutory health insurance "AOK—Die Gesundheitskasse"
ATC: Anatomical Therapeutic Chemical classification
CCI: Charlson comorbidity index
CI: Confidence interval
DDD: Defined daily doses
DNA: Deoxyribonucleic acid
EMA: Europeans Medicines Agency
hERG: Human Ether-a-go-go-related Gene
ICD-10-GM: German modification of International Statistical Classification of Diseases and Related Health Problems 10th version
IR: Incidence rate
OPS: German adaptation of the International Classification of Procedures in Medicine
PAMM: Piece-wise exponential additive mixed models
PS: Propensity score
US: United States
WHO: World Health Organization
