# Supplementary material for: High risk for life-threatening adverse events of fluoroquinolones in young adults: a large German population-based cohort study
Source: BMC Med. 2025 Feb 7;23:76. doi: 10.1186/s12916-025-03919-0 (PMC11806691; doi:10.1186/s12916-025-03919-0)
Supplement: Supplementary file 1 — Additional File 1. Table S1. Definition of study variables. Figure S1. Cohort attrition. Table S2a. Study population characteristics, cohort 1-2. Table S2b. Study population characteristics, cohort 3-4. Figure S2. Smooth plots from PAMM regression models, cohort 1-4. Table S3. Age- and gender-standardized incidence rate (per 10,000 episodes), stratified by age groups, cohort 1-4. Table S4a. Study population characteristics, 1:1 PS matched cohort 1-2. Table S4b. Study population characteristics, 1:1 PS matched cohort 3-4. Table S5. Results from PAMM regression models, PS-matched cohorts. Table S6. Results from PAMM regression models in sensitivity analyses, cohort 1-4 [file 12916_2025_3919_MOESM1_ESM.docx]

**Additional File 1:**

Table S1. Definition of study variables

Figure S1. Cohort attrition

Table S2a. Study population characteristics, cohort 1-2

Table S2b. Study population characteristics, cohort 3-4

Figure S2. Smooth plots from PAMM regression models, cohort 1-4

Table S3. Age- and gender-standardized incidence rate (per 10,000 episodes), stratified by age groups, cohort 1-4

Table S4a. Study population characteristics, 1:1 PS matched cohort 1-2

Table S4b. Study population characteristics, 1:1 PS matched cohort 3-4

Table S5. Results from PAMM regression models, PS-matched cohorts

Table S6. Results from PAMM regression models in sensitivity analyses, cohort 1-4

Table S1. Definition of study variables

| **Cohort** | **Variable** | **Definition** | |
| --- | --- | --- | --- |
| All | Exposure | ATC^1^: | J01MA |
|  | Active comparators | ATC^1^: | J01CA04, J01DC02, J01FA10, J01FF01, J01CR02, J01EE01, J01DB01, J01AA02 |
|  | Covariates:  Age  Gender  Charlson Comorbidity Index (CCI)  Drugs dispensed  Hospitalized days  Year of cohort entry date  Quarter of cohort entry date | ICD^2^:  ATC^1^: | Date of birth  Self-reported gender registered at AOK  Modified/Updated definition used by Quan et al. [15]  Counts of ATC codes per day  Counts of hospitalized days  Year of cohort entry date (i.e. derived from date of index antibiotic prescription dispensed)  Quarter of cohort entry date (i.e. derive from date of index antibiotic prescription dispensed) |
| Endpoint-specific |  |  |  |
| Aortic aneurysm/dissection | Outcome of interest | ICD^2^: | I71 |
|  | Covariates:  Cardiovascular diseases (CVD) | ICD^2^: | I200, I21, I22, I119, I201, I208, I209, I24, I25, I110, I130, I132, I42, I43, I50, J81, I34, I35, I36, I37, G45, G46, I60, I61, I62, I63, I64, I65, I66, I67, I68, I69, I70, I72, I73, I74, I77, K550, K551, I44, I45, I46, I47, I48, I49 |
|  | Cardiac surgery | OPS^3^: | 5-35, 5-36, 5-37 |
|  | Diabetes mellitus | ICD^2^: | E10, E11, E12, E13, E14 |
| Cardiac arrhythmia/sudden cardiac death | Outcome of interest | ICD^2^: | I44-I49 |
|  | Covariates:  CVD | ICD^2^: | I200, I21, I22, I119, I201, I208, I209, I24, I25, I110, I130, I132, I42, I43, I50, J81, I34, I35, I36, I37, G45, G46, I60, I61, I62, I63, I64, I65, I66, I67, I68, I69, I70, I71, I72, I73, I74, I77, K550, K551 |
|  | Renal diseases | ICD^2^: | I12, I13, N00, N01, N02, N03, N04, N05, N06, N07, N08, N17, N18, N19, N25, Z49, Z940, Z992 |
|  | Chronic lower respiratory diseases (COPD) | ICD^2^: | J40, J41, J42, J43, J44, J45, J46, J47 |
|  | Diabetes mellitus | ICD^2^: | E10, E11, E12, E13, E14 |
| Acute toxic liver injury/acute liver failure | Outcome of interest | ICD^2^: | K71.0, K71.1, K71.2, K71.6, K71.8, K71.9, K72.0, K72.9 |
|  | Covariates:  Renal diseases | ICD^2^: | I12, I13, N00, N01, N02, N03, N04, N05, N06, N07, N08, N17, N18, N19, N25, Z49, Z940, Z992 |
|  | Liver disease | ICD^2^: | B15, B16, B17, B18, B19, K70, K713, K715, K717, K721, K73, K74, K75, K76, K770, K778, R160, R162, R17, R932, R945 |
|  | Diabetes mellitus | ICD^2^: | E10, E11, E12, E13, E14 |
| All-cause mortality | Outcome of interest |  | Date of death |
| 1 Anatomic-Therapeutic-Chemical (ATC) classification, the German adaptation of WHO-ATC classification, version 2020  2 International Classification of Diseases, 10th revision, German modification (ICD-10-GM), version 2020  3 “*Operationen- und Prozedurenschlüssel*” (OPS), the German adaptation of the International Classification of Procedures in Medicine, version 2020 | | | |


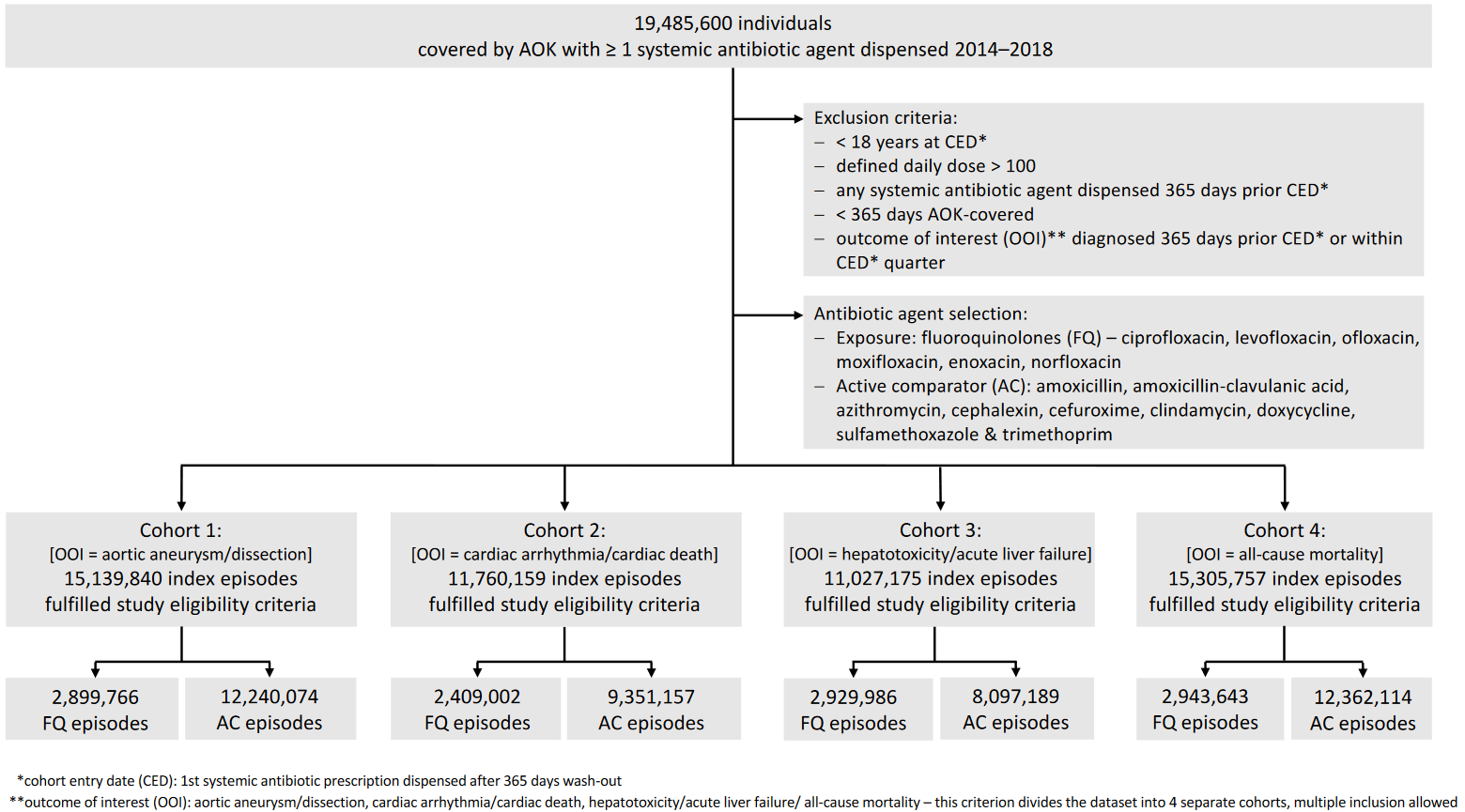


Figure S1. Cohort attrition.

Table 2a. Study population characteristics, cohort 1-2

|  | **Cohort 1: aortic aneurysm/dissection** | | |  | **Cohort 2: cardiac arrhythmia/sudden cardiac death** | | |
| --- | --- | --- | --- | --- | --- | --- | --- |
|  | **Fluoroquinolones** | **Active comparators** | **Standardised difference** |  | **Fluoroquinolones** | **Active comparators** | **Standardised difference** |
|  | **(n= 2,899,766)** | **(n= 12,240,074)** |  |  | **(n= 2,409,002)** | **(n= 9,351,157)** |  |
| Age (mean (SD)) | 59.29 (19.48) | 51.08 (19.49) | 0.422 |  | 56.29 (19.10) | 49.20 (18.62) | 0.376 |
| Male gender (%) | 1,214,636 (41.89) | 5,604,170 (45.79) | 0.079 |  | 1,006,643 (41.79) | 4,325,235 (46.25) | 0.090 |
| CCI (%) |  |  | 0.244 |  |  |  | 0.159 |
| 0 | 1,552,033 (53.52) | 7,865,625 (64.26) |  |  | 1,470,360 (61.04) | 6,463,710 (69.12) |  |
| 1-2 | 897,726 (30.96) | 3,230,746 (26.40) |  |  | 688,937 (28.60) | 2,278,262 (24.36) |  |
| 3-4 | 311,965 (10.76) | 843,986 (6.90) |  |  | 173,718 (7.21) | 454,080 (4.86) |  |
| 5+ | 138,042 (4.76) | 299,717 (2.45) |  |  | 75,987 (3.15) | 155,105 (1.66) |  |
| Drugs dispensed (%) |  |  | 0.330 |  |  |  | 0.287 |
| 0 | 357,395 (12.33) | 2,368,866 (19.35) |  |  | 349,217 (14.50) | 1,985,193 (21.23) |  |
| 1-3 | 498,020 (17.17) | 2,864,771 (23.41) |  |  | 477,883 (19.84) | 2,345,966 (25.09) |  |
| 4-10 | 638,946 (22.03) | 2,834,113 (23.15) |  |  | 580,155 (24.08) | 2,243,232 (23.99) |  |
| 11-20 | 533,665 (18.40) | 1,820,589 (14.87) |  |  | 430,852 (17.89) | 1,329,873 (14.22) |  |
| 21+ | 871,740 (30.06) | 2,351,735 (19.21) |  |  | 570,895 (23.70) | 1,446,893 (15.47) |  |
| Hospitalised days (%) |  |  | 0.213 |  |  |  | 0.162 |
| 0 | 2,131,158 (73.49) | 9,960,780 (81.38) |  |  | 1,875,081 (77.84) | 7,793,398 (83.34) |  |
| 1-7 | 372,026 (12.83) | 1,342,196 (10.97) |  |  | 287,052 (11.92) | 983,229 (10.52) |  |
| 8+ | 396,582 (13.68) | 937,098 (7.66) |  |  | 246,869 (10.25) | 574,530 (6.14) |  |
| Cohort entry year (%) |  |  | 0.142 |  |  |  | 0.138 |
| 2014 | 651,480 (22.47) | 2,342,227 (19.14) |  |  | 542,974 (22.54) | 1,811,910 (19.38) |  |
| 2015 | 635,397 (21.91) | 2,405,549 (19.65) |  |  | 527,945 (21.92) | 1,834,869 (19.62) |  |
| 2016 | 598,715 (20.65) | 2,446,280 (19.99) |  |  | 496,274 (20.60) | 1,868,365 (19.98) |  |
| 2017 | 535,284 (18.46) | 2,488,135 (20.33) |  |  | 443,538 (18.41) | 1,892,449 (20.24) |  |
| 2018 | 478,890 (16.52) | 2,557,883 (20.90) |  |  | 398,271 (16.53) | 1,943,564 (20.78) |  |
| Cohort entry quarter (%) |  |  | 0.077 |  |  |  | 0.052 |
| Q1 (Jan.-Mar.) | 891,565 (30.75) | 4,049,558 (33.08) |  |  | 740,377 (30.73) | 3,003,834 (32.12) |  |
| Q2 (Apr.-Jun.) | 643,880 (22.21) | 2,643,994 (21.60) |  |  | 532,623 (22.11) | 2,058,191 (22.01) |  |
| Q3 (Jul.-Sep.) | 650,252 (22.42) | 2,408,277 (19.68) |  |  | 540,931 (22.46) | 1,910,116 (20.43) |  |
| Q4 (Oct.-Dec.) | 714,069 (24.63) | 3,138,245 (25.64) |  |  | 595,071 (24.70) | 2,379,016 (25.44) |  |
| Outcome-specific additional variables | |  |  |  |  |  |  |
| Diabetes mellitus (%) | 679,175 (23.42) | 1,949,067 (15.92) | 0.189 |  | 477,985 (19.84) | 1,299,640 (13.90) | 0.159 |
| CVD (%) | 1,152,509 (39.75) | 3,319,243 (27.12) | 0.270 |  | 712,550 (29.58) | 1,893,575 (20.25) | 0.217 |
| Cardiac surgery (%) | 20,973 (0.72) | 59,564 (0.49) | 0.031 |  | *NA* | *NA* |  |
| COPD (%) | *NA* | *NA* |  |  | 470,389 (19.53) | 1,609,440 (17.21) | 0.060 |
| Renal diseases (%) | *NA* | *NA* |  |  | 246,349 (10.23) | 555,228 (5.94) | 0.158 |
| CCI = Charlson comorbidity index \| CED = cohort entry date \| Q = quarter \| CVD = cardiovascular diseases \| COPD = chronic obstructive pulmonary diseases \| NA = not applicable. \| health status-related variables = CCI, drugs dispensed, and hospitalized days \| outcome-specific additional variables = comorbidities associated with the respective outcome of interest. | | | | | | | |

Table 2b. Study population characteristics, cohort 3-4

|  | **Cohort 3: Acute toxic liver injury/acute liver failure** | | |  | **Cohort 4: all-cause mortality** | | |
| --- | --- | --- | --- | --- | --- | --- | --- |
|  | **Fluoroquinolones** | **Active comparators** | **Standardised difference** |  | **Fluoroquinolones** | **Active comparators** | **Standardised difference** |
|  | **(n= 2 929 986)** | **(n= 8 097 189)** |  |  | **(n= 2 943 643)** | **(n= 12 362 114)** |  |
| Age (mean SD) | 59.49 (19.47) | 50.56 (19.49) | 0.459 |  | 59.52 (19.45) | 51.28 (19.54) | 0.423 |
| Male sex (%) | 1,237,945 (42.25) | 3,711,594 (45.84) | 0.072 |  | 1,245,739 (42.32) | 5,692,100 (46.05) | 0.075 |
| CCI (%) |  |  | 0.224 |  |  |  | 0.212 |
| 0 | 1,830,144 (62.46) | 5,818,473 (71.86) |  |  | 1,836,477 (62.39) | 8,819,353 (71.34) |  |
| 1-2 | 849,127 (28.98) | 1,915,470 (23.66) |  |  | 854,058 (29.01) | 2,955,802 (23.91) |  |
| 3-4 | 166,830 (5.69) | 256,649 (3.17) |  |  | 168,112 (5.71) | 411,825 (3.33) |  |
| 5+ | 83,885 (2.86) | 106,597 (1.32) |  |  | 84,996 (2.89) | 175,134 (1.42) |  |
| Drugs dispensed (%) |  |  | 0.362 |  |  |  | 0.331 |
| 0 | 357,406 (12.20) | 1,590,598 (19.64) |  |  | 357,850 (12.16) | 2,370,621 (19.18) |  |
| 1-3 | 498,368 (17.01) | 1,935,846 (23.91) |  |  | 499,358 (16.96) | 2,869,495 (23.21) |  |
| 4-10 | 642,041 (21.91) | 1,891,800 (23.36) |  |  | 644,235 (21.89) | 2,851,536 (23.07) |  |
| 11-20 | 541,446 (18.48) | 1,192,136 (14.72) |  |  | 544,293 (18.49) | 1,851,903 (14.98) |  |
| 21+ | 890,725 (30.40) | 1,486,809 (18.36) |  |  | 897,907 (30.50) | 2,418,559 (19.56) |  |
| Hospitalised days (%) |  |  | 0.249 |  |  |  | 0.215 |
| 0 | 2,146,648 (73.27) | 6,690,917 (82.63) |  |  | 2,152,973 (73.14) | 10,031,108 (81.14) |  |
| 1-7 | 378,030 (12.90) | 835,393 (10.32) |  |  | 379,894 (12.91) | 1,363,989 (11.03) |  |
| 8+ | 405,308 (13.83) | 570,879 (7.05) |  |  | 410,776 (13.96) | 967,017 (7.82) |  |
| Cohort entry year (%) |  |  | 0.166 |  |  |  | 0.141 |
| 2014 | 657,023 (22.42) | 1,513,472 (18.69) |  |  | 660,241 (22.43) | 2,362,729 (19.11) |  |
| 2015 | 641,317 (21.89) | 1,556,315 (19.22) |  |  | 644,244 (21.89) | 2,428,127 (19.64) |  |
| 2016 | 605,032 (20.65) | 1,596,258 (19.71) |  |  | 607,853 (20.65) | 2,470,390 (19.98) |  |
| 2017 | 541,462 (18.48) | 1,658,545 (20.48) |  |  | 543,963 (18.48) | 2,514,282 (20.34) |  |
| 2018 | 485,152 (16.56) | 1,772,599 (21.89) |  |  | 487,342 (16.56) | 2,586,586 (20.92) |  |
| Cohort entry quarter (%) |  |  | 0.096 |  |  |  | 0.077 |
| Q1 (Jan.-Mar.) | 900,433 (30.73) | 2,729,165 (33.71) |  |  | 904,490 (30.73) | 4,089,357 (33.08) |  |
| Q2 (Apr.-Jun.) | 650,809 (22.21) | 1,715,442 (21.19) |  |  | 654,055 (22.22) | 2,670,842 (21.61) |  |
| Q3 (Jul.-Sep.) | 657,219 (22.43) | 1,550,309 (19.15) |  |  | 660,250 (22.43) | 2,431,864 (19.67) |  |
| Q4 (Oct.-Dec.) | 721,525 (24.63) | 2,102,273 (25.96) |  |  | 724,848 (24.62) | 3,170,051 (25.64) |  |
| Outcome-specific additional variables | |  |  |  |  |  |  |
| Diabetes mellitus (%) | 689,470 (23.53) | 1,238,506 (15.30) | 0.209 |  | *NA* | *NA* |  |
| Liver diseases (%) | 347,260 (11.85) | 744,091 (9.19) | 0.087 |  | *NA* | *NA* |  |
| Renal diseases (%) | 427,753 (14.60) | 627,514 (7.75) | 0.219 |  | *NA* | *NA* |  |
| CCI = Charlson comorbidity index \| CED = cohort entry date \| Q = quarter \| NA = not applicable. \| health status-related variables = CCI, drugs dispensed, and hospitalized days \| Outcome-specific additional variables = comorbidities associated with the respective outcome of interest. | | | | | | | |

| **Cohort 1:  aortic aneurysm/dissection** | **Cohort 2:**  **cardiac arrhythmia/sudden cardiac death** | **Cohort 3: acute toxic liver injury / acute liver failure** | **Cohort 4:  all-cause mortality** |
| --- | --- | --- | --- |
| 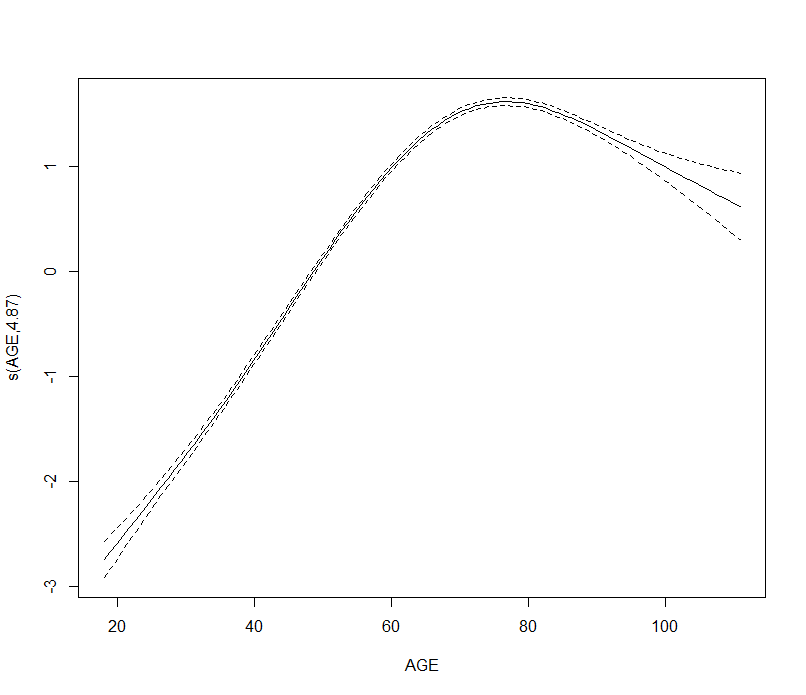 | 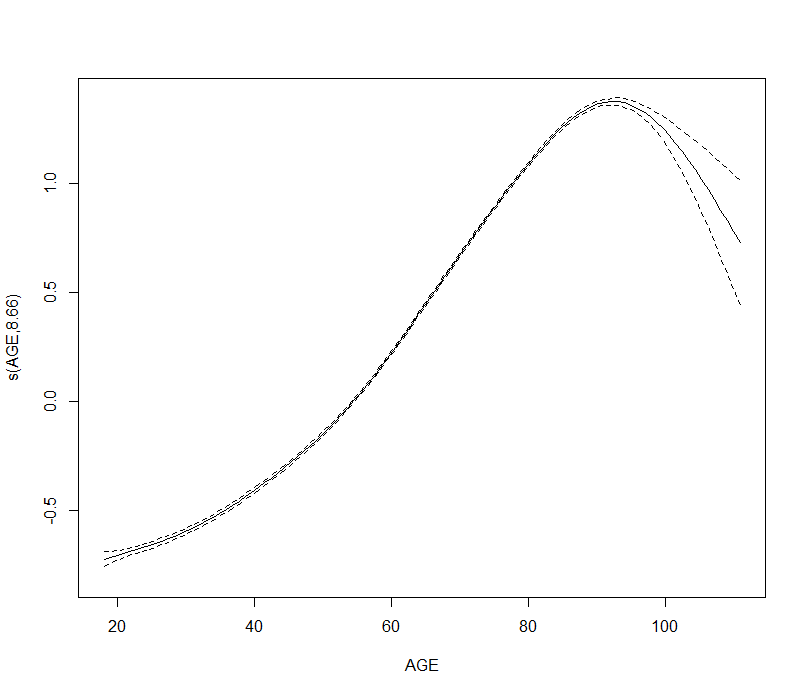 | 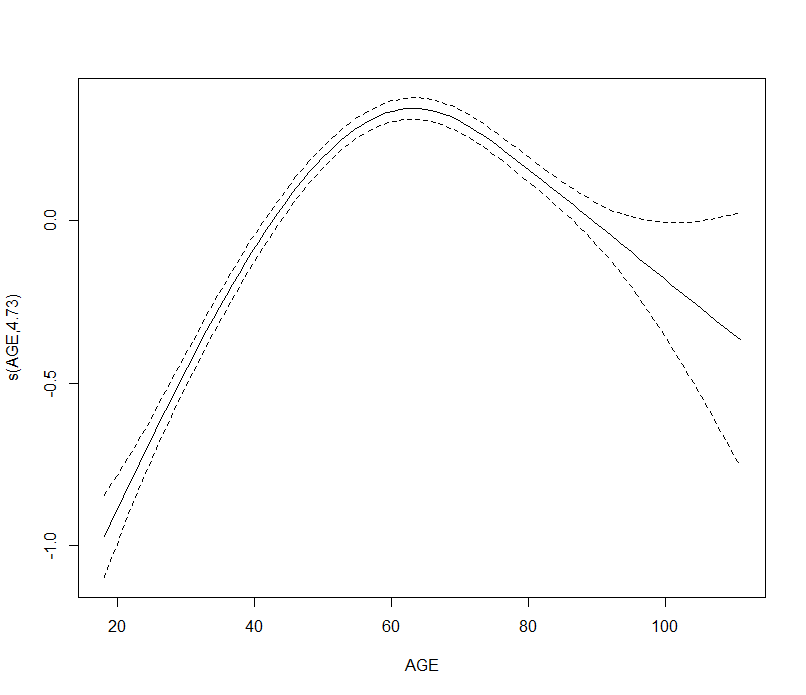 | 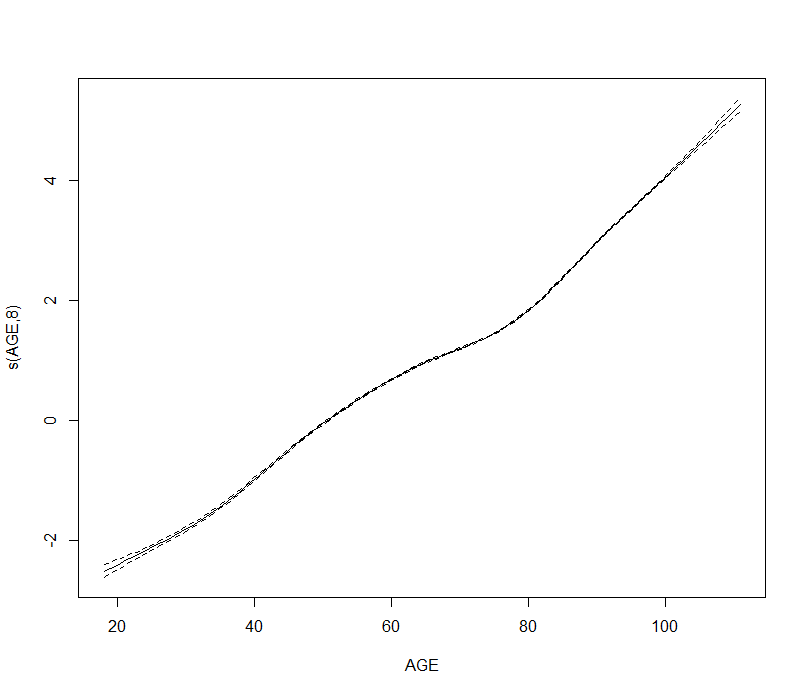 |
| 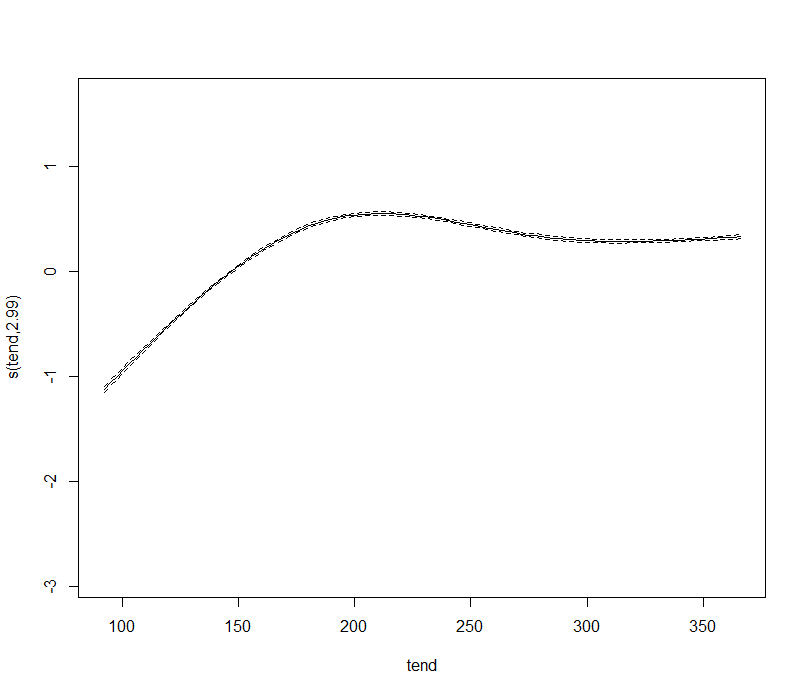 | 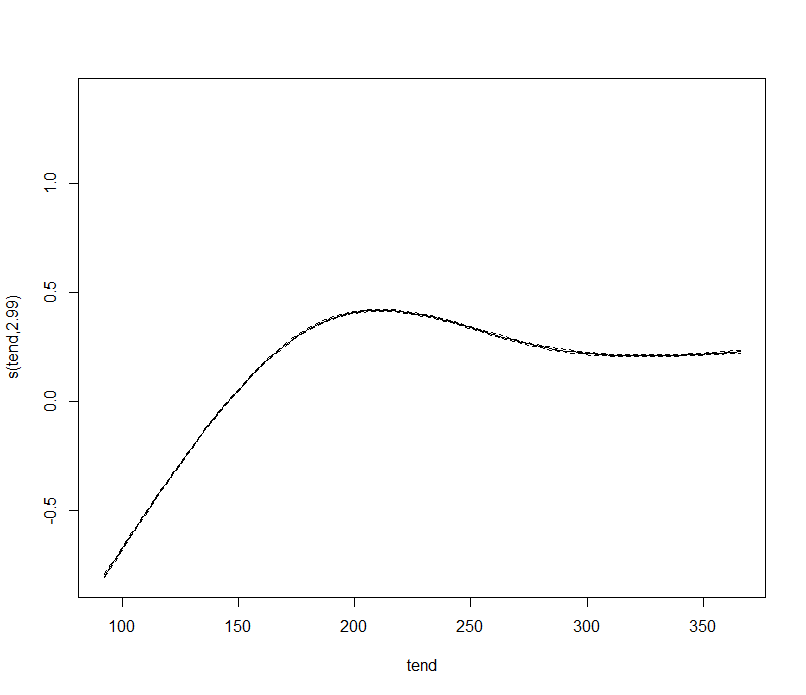 | 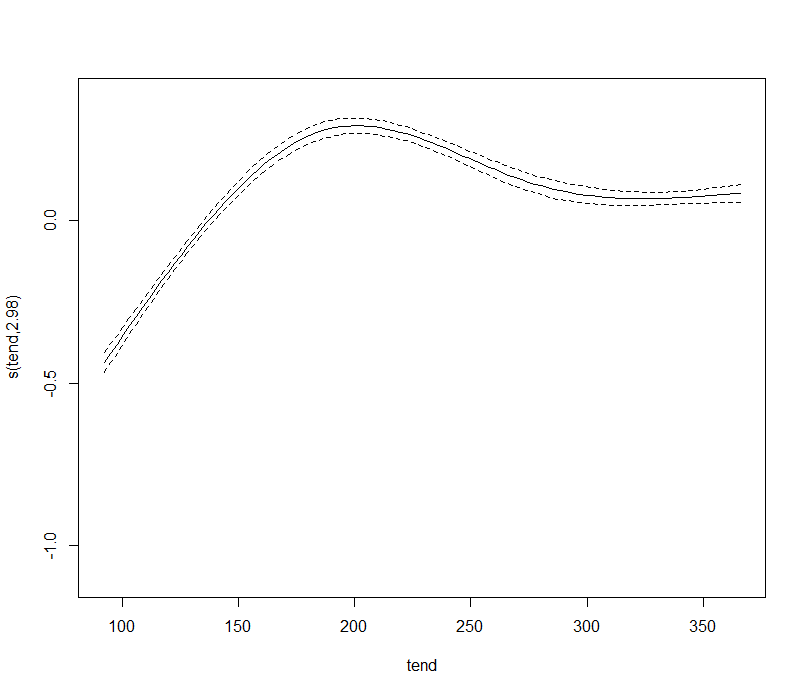 | 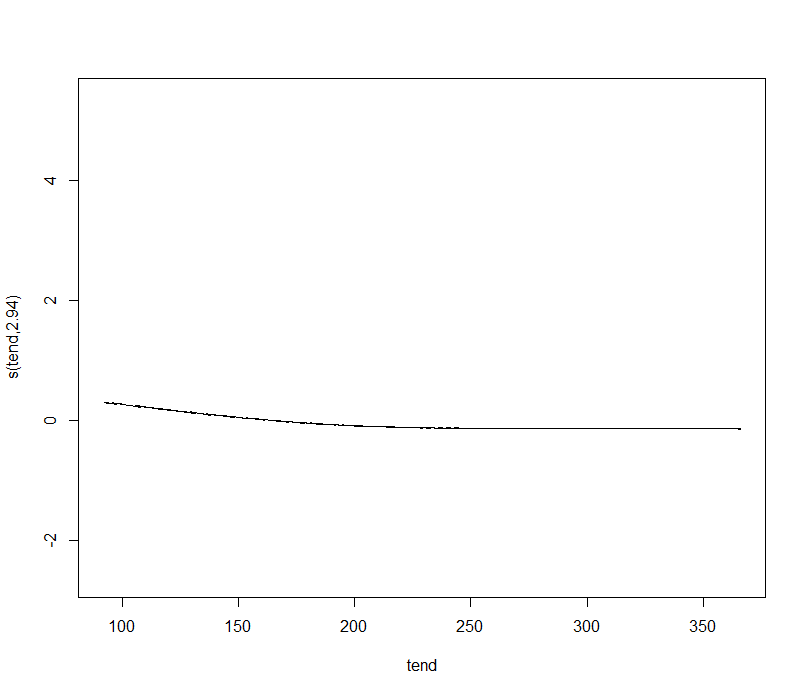 |
| AGE = age at cohort entry date in years \| t_end_ = individuals’ person time of follow-up. | | | |

Figure S2. Smooth plots from PAMM regression models, cohort 1-4

Table S3. Age- and gender-standardized incidence rate (per 10,000 episodes), stratified by age groups, cohort 1-4

| **Outcome of interest** | **Age group** | **Fluoroquinolones** | | **Active comparators** | |
| --- | --- | --- | --- | --- | --- |
|  |  | **sIR** | **[95% CI]** | **sIR** | **[95% CI]** |
| Aortic events | 18-39 years | 2 | [1;3] | 1 | [1;2] |
|  | 40-69 years | 25 | [22;27] | 23 | [22;25] |
|  | ≥ 70 years | 88 | [83;94] | 86 | [82;90] |
| Cardiac events | 18-39 years | 116 | [106;126] | 101 | [98;105] |
|  | 40-69 years | 296 | [286;307] | 271 | [266;276] |
|  | ≥ 70 years | 1,061 | [1,036;1,086] | 1,022 | [1,006;1,039] |
| Hepatic events | 18-39 years | 6 | [3;8] | 4 | [3;5] |
|  | 40-69 years | 23 | [20;25] | 14 | [13;15] |
|  | ≥ 70 years | 36 | [33;40] | 27 | [24;29] |
| All-cause mortality | 18-39 years | 13 | [10;16] | 6 | [5;7] |
|  | 40-69 years | 183 | [175;190] | 90 | [88;93] |
|  | ≥ 70 years | 1,290 | [1,267;1,312] | 974 | [961;987] |
| sIR = age- and gender-standardized incidence rate (German Census 2011) per 10,000 index episodes, cases presented are rounded to whole numbers  95% CI = lower and upper bound of 95% confidence interval | | | | | |

Table S4a. Study population characteristics, 1:1 PS matched cohort 1-2

|  | **Cohort 1: aortic aneurysm/dissection** | | |  | **Cohort 2: cardiac arrhythmia/sudden cardiac death** | | |
| --- | --- | --- | --- | --- | --- | --- | --- |
|  | **Fluoroquinolones** | **Active comparators** | **Standardised difference** |  | **Fluoroquinolones** | **Active comparators** | **Standardised difference** |
|  | **(n= 2 899 766)** | **(n= 2 899 766)** |  |  | **(n= 2 409 001)** | **(n= 2 409 001)** |  |
| Age (mean (SD)) | 59.29 (19.48) | 59.32 (19.47) | 0.001 |  | 56.29 (19.10) | 56.39 (19.14) | 0.005 |
| Male gender (%) | 1,214,636 (41.89) | 1,208,598 (41.68) | 0.004 |  | 1,006,643 (41.79) | 993,264 (41.23) | 0.011 |
| CCI (%) |  |  | 0.010 |  |  |  | 0.002 |
| 0 | 1,552,033 (53.52) | 1,555,009 (53.63) |  |  | 1,470,360 (61.04) | 1,477,046 (61.31) |  |
| 1-2 | 897,726 (30.96) | 901,619 (31.09) |  |  | 688,937 (28.60) | 695,561 (28.87) |  |
| 3-4 | 311,965 (10.76) | 310,833 (10.72) |  |  | 173,718 (7.21) | 168,789 (7.01) |  |
| 5+ | 138,042 (4.76) | 132,305 (4.56) |  |  | 75,986 (3.15) | 67,605 (2.81) |  |
| Drugs dispensed (%) |  |  | 0.006 |  |  |  | 0.008 |
| 0 | 357,395 (12.33) | 354,752 (12.23) |  |  | 349,217 (14.50) | 347,021 (14.41) |  |
| 1-3 | 498,020 (17.17) | 494,865 (17.07) |  |  | 477,883 (19.84) | 474,430 (19.69) |  |
| 4-10 | 638,946 (22.03) | 636,395 (21.95) |  |  | 580,155 (24.08) | 578,124 (24.00) |  |
| 11-20 | 533,665 (18.40) | 534,223 (18.42) |  |  | 430,852 (17.89) | 430,882 (17.89) |  |
| 21+ | 871,740 (30.06) | 879,531 (30.33) |  |  | 570,894 (23.70) | 578,544 (24.02) |  |
| Hospitalised days (%) |  |  | 0.007 |  |  |  | 0.016 |
| 0 | 2,131,158 (73.49) | 2,139,423 (73.78) |  |  | 1,875,081 (77.84) | 1,889,892 (78.45) |  |
| 1-7 | 372,026 (12.83) | 369,854 (12.76) |  |  | 287,052 (11.92) | 281,806 (11.70) |  |
| 8+ | 396,582 (13.68) | 390,489 (13.47) |  |  | 246,868 (10.25) | 237,303 (9.85) |  |
| Cohort entry year (%) |  |  | 0.001 |  |  |  | 0.002 |
| 2014 | 651,480 (22.47) | 651,538 (22.47) |  |  | 542,973 (22.54) | 543,714 (22.57) |  |
| 2015 | 635,397 (21.91) | 636,138 (21.94) |  |  | 527,945 (21.92) | 528,057 (21.92) |  |
| 2016 | 598,715 (20.65) | 598,570 (20.64) |  |  | 496,274 (20.60) | 497,107 (20.64) |  |
| 2017 | 535,284 (18.46) | 535,625 (18.47) |  |  | 443,538 (18.41) | 443,620 (18.42) |  |
| 2018 | 478,890 (16.52) | 477,895 (16.48) |  |  | 398,271 (16.53) | 396,503 (16.46) |  |
| Cohort entry quarter (%) |  |  | 0.002 |  |  |  | 0.003 |
| Q1 (Jan.-Mar.) | 891,565 (30.75) | 893,068 (30.80) |  |  | 740,377 (30.73) | 742,158 (30.81) |  |
| Q2 (Apr.-Jun.) | 643,880 (22.21) | 643,748 (22.20) |  |  | 532,623 (22.11) | 530,801 (22.03) |  |
| Q3 (Jul.-Sep.) | 650,252 (22.42) | 648,523 (22.37) |  |  | 540,930 (22.46) | 539,686 (22.40) |  |
| Q4 (Oct.-Dec.) | 714,069 (24.63) | 714,427 (24.64) |  |  | 595,071 (24.70) | 596,356 (24.76) |  |
| Outcome-specific additional covariables | |  |  |  |  |  |  |
| Diabetes mellitus (%) | 679,175 (23.42) | 676,611 (23.33) | 0.002 |  | 477,985 (19.84) | 475,981 (19.76) | 0.002 |
| CVD (%) | 1,152,509 (39.75) | 1,154,315 (39.81) | 0.001 |  | 712,549 (29.58) | 718,480 (29.83) | 0.005 |
| Cardiac surgery (%) | 20,973 (0.72) | 17,638 (0.61) | 0.014 |  | *NA* | *NA* |  |
| COPD (%) | *NA* | *NA* |  |  | 470,389 (19.53) | 468,975 (19.47) | 0.001 |
| Renal diseases (%) | *NA* | *NA* |  |  | 246,348 (10.23) | 235,741 (9.79) | 0.015 |
| CCI = Charlson comorbidity index \| Q = quarter \| CVD = cardiovascular diseases \| COPD = chronic lower respiratory diseases \| NA = not applicable. \| health status-related variables = CCI, drugs dispensed, and hospitalized days \| Outcome-specific additional variables = comorbidities associated with the respective outcome of interest. | | | | | | | |

Table S4b. Study population characteristics, 1:1 Propensity score matched cohort 3-4

|  | **Cohort 3: Acute toxic liver injury/acute liver failure** | | |  | **Cohort 4: all-cause mortality** | | |
| --- | --- | --- | --- | --- | --- | --- | --- |
|  | **Fluoroquinolones** | **Active comparators** | **Standardised difference** |  | **Fluoroquinolones** | **Active comparators** | **Standardised difference** |
|  | **(n= 71 572)** | **(n= 71 572)** |  |  | **(n= 2 943 643)** | **(n= 2 943 643)** |  |
| Age (mean SD) | 59.76 (19.41) | 59.94 (19.54) | 0.009 |  | 59.52 (19.45) | 59.53 (19.45) | 0.001 |
| Male sex (%) | 30,062 (42.00) | 28,929 (40.42) | 0.032 |  | 1,245,739 (42.32) | 1,244,291 (42.27) | 0.001 |
| CCI (%) |  |  | 0.026 |  |  |  | 0.005 |
| 0 | 44,567 (62.27) | 45,198 (63.15) |  |  | 1,836,477 (62.39) | 1,838,172 (62.45) |  |
| 1-2 | 20,594 (28.77) | 20,436 (28.55) |  |  | 854,058 (29.01) | 855,322 (29.06) |  |
| 3-4 | 4,278 (5.98) | 4,000 (5.59) |  |  | 168,112 (5.71) | 167,407 (5.69) |  |
| 5+ | 2,133 (2.98) | 1,938 (2.71) |  |  | 84,996 (2.89) | 82,742 (2.81) |  |
| Drugs dispensed (%) |  |  | 0.014 |  |  |  | 0.003 |
| 0 | 8,595 (12.01) | 8,487 (11.86) |  |  | 357,850 (12.16) | 356,283 (12.10) |  |
| 1-3 | 12,034 (16.81) | 12,032 (16.81) |  |  | 499,358 (16.96) | 497,851 (16.91) |  |
| 4-10 | 15,557 (21.74) | 15,933 (22.26) |  |  | 644,235 (21.89) | 644,259 (21.89) |  |
| 11-20 | 13,358 (18.66) | 13,344 (18.64) |  |  | 544,293 (18.49) | 544,849 (18.51) |  |
| 21+ | 22,028 (30.78) | 21,776 (30.43) |  |  | 897,907 (30.50) | 900,401 (30.59) |  |
| Hospitalised days (%) |  |  | 0.035 |  |  |  | 0.002 |
| 0 | 52,335 (73.12) | 53,418 (74.64) |  |  | 2,152,973 (73.14) | 2,155,289 (73.22) |  |
| 1-7 | 9,191 (12.84) | 8,723 (12.19) |  |  | 379,894 (12.91) | 379,585 (12.90) |  |
| 8+ | 10,046 (14.04) | 9,431 (13.18) |  |  | 410,776 (13.96) | 408,769 (13.89) |  |
| Cohort entry year (%) |  |  | 0.008 |  |  |  | <0.001 |
| 2014 | 16,131 (22.54) | 16,162 (22.58) |  |  | 660,241 (22.43) | 660,052 (22.42) |  |
| 2015 | 15,718 (21.96) | 15,739 (21.99) |  |  | 644,244 (21.89) | 644,470 (21.89) |  |
| 2016 | 15,062 (21.05) | 15,227 (21.28) |  |  | 607,853 (20.65) | 607,985 (20.65) |  |
| 2017 | 13,064 (18.25) | 13,020 (18.19) |  |  | 543,963 (18.48) | 543,962 (18.48) |  |
| 2018 | 11,597 (16.20) | 11,424 (15.96) |  |  | 487,342 (16.56) | 487,174 (16.55) |  |
| Cohort entry quarter (%) |  |  | 0.009 |  |  |  | <0.001 |
| Q1 (Jan.-Mar.) | 21,952 (30.67) | 22,020 (30.77) |  |  | 904,490 (30.73) | 904,765 (30.74) |  |
| Q2 (Apr.-Jun.) | 15,929 (22.26) | 16,058 (22.44) |  |  | 654,055 (22.22) | 653,740 (22.21) |  |
| Q3 (Jul.-Sep.) | 16,067 (22.45) | 15,814 (22.10) |  |  | 660,250 (22.43) | 660,215 (22.43) |  |
| Q4 (Oct.-Dec.) | 17,624 (24.62) | 17,680 (24.70) |  |  | 724,848 (24.62) | 724,923 (24.63) |  |
| Outcome-specific additional covariables | |  |  |  |  |  |  |
| Diabetes mellitus (%) | 17,028 (23.79) | 16,447 (22.98) | 0.019 |  | *NA* | *NA* |  |
| Liver diseases (%) | 8,418 (11.76) | 7,723 (10.79) | 0.031 |  | *NA* | *NA* |  |
| Renal diseases (%) | 10,569 (14.77) | 10,067 (14.07) | 0.020 |  | *NA* | *NA* |  |
| CCI = Charlson comorbidity index \| Q = quarter \| NA = not applicable. \| health status-related variables = CCI, drugs dispensed, and hospitalized days \| Outcome-specific additional variables = comorbidities associated with the respective outcome of interest. | | | | | | | |

Table S5. Results from PAMM regression models, Propensity score matched cohorts

|  | **Cohort 1:** | |  | **Cohort 2:** | |  | **Cohort 3:** | |  | **Cohort 4:** | |
| --- | --- | --- | --- | --- | --- | --- | --- | --- | --- | --- | --- |
|  | **aortic aneurysm/dissection** | |  | **cardiac arrhythmia/sudden cardiac death** | |  | **acute toxic liver injury / acute liver failure** | |  | **all-cause mortality** | |
|  | **aHR** | **[95% CI]** |  | **aHR** | **[95% CI]** |  | **aHR** | **[95% CI]** |  | **aHR** | **[95% CI]** |
| Fluoroquinolone episode (ref. active comparators) | 1.062 | [1.032;1.093] |  | 1.041 | [1.032;1.051] |  | 1.442 | [1.139;1.826] |  | 1.219 | [1.210;1.228] |
| Males (ref. females) | 2.924 | [2.833;3.017] |  | 1.276 | [1.264;1.288] |  | 1.576 | [1.245;1.995] |  | 1.438 | [1.428;1.449] |
| CCI (ref. 0) |  |  |  |  |  |  |  | |  |  | |
| 1-2 | 1.100 | [1.061;1.139] |  | 0.957 | [0.946;0.968] |  | 1.252 | [0.954;1.644] |  | 1.708 | [1.692;1.725] |
| 3-4 | 1.085 | [1.037;1.135] |  | 0.946 | [0.930;0.962] |  | 1.054 | [0.682;1.630] |  | 2.285 | [2.259;2.313] |
| 5+ | 0.987 | [0.928;1.050] |  | 1.020 | [0.997;1.042] |  | 2.215 | [1.465;3.348] |  | 4.638 | [4.580;4.696] |
| Drugs dispensed (ref. 0) |  |  |  |  |  |  |  | |  |  | |
| 1-3 | 1.066 | [0.962;1.180] |  | 1.158 | [1.127;1.190] |  | 1.156 | [0.480;2.782] |  | 0.831 | [0.796;0.869] |
| 4-10 | 1.168 | [1.064;1.281] |  | 1.402 | [1.367;1.438] |  | 1.623 | [0.725;3.636] |  | 1.022 | [0.984;1.062] |
| 11-20 | 1.325 | [1.207;1.455] |  | 1.622 | [1.580;1.665] |  | 2.298 | [1.025;5.151] |  | 1.298 | [1.250;1.347] |
| 21+ | 1.381 | [1.256;1.518] |  | 2.084 | [2.030;2.140] |  | 3.475 | [1.556;7.760] |  | 2.118 | [2.042;2.197] |
| Hospitalised days (ref. 0) |  |  |  |  |  |  |  | |  |  | |
| 1-7 | 1.057 | [1.015;1.101] |  | 1.007 | [0.994;1.021] |  | 1.655 | [1.165;2.352] |  | 1.331 | [1.316;1.347] |
| 8+ | 1.056 | [1.016;1.098] |  | 1.088 | [1.074;1.102] |  | 3.332 | [2.504;4.434] |  | 2.482 | [2.461;2.503] |
| Cohort entry year (ref. 2014) |  |  |  |  |  |  |  | |  |  | |
| 2015 | 1.005 | [0.960;1.051] |  | 0.983 | [0.970;0.997] |  | 1.578 | [1.109;2.246] |  | 0.957 | [0.946;0.967] |
| 2016 | 1.043 | [0.996;1.091] |  | 0.992 | [0.979;1.006] |  | 1.077 | [0.731;1.585] |  | 0.974 | [0.963;0.985] |
| 2017 | 1.136 | [1.085;1.189] |  | 0.945 | [0.931;0.958] |  | 1.240 | [0.843;1.824] |  | 0.961 | [0.950;0.972] |
| 2018 | 1.273 | [1.216;1.332] |  | 0.937 | [0.923;0.951] |  | 1.409 | [0.960;2.069] |  | 0.945 | [0.934;0.956] |
| Cohirt entry quarter (ref. Q1 (Jan.-Mar.) | |  |  |  |  |  |  | |  |  | |
| Q2 (Apr.-Jun.) | 1.043 | [1.002;1.086] |  | 1.021 | [1.009;1.034] |  | 1.073 | [0.782;1.473] |  | 1.034 | [1.023;1.044] |
| Q3 (Jul.-Sep.) | 1.074 | [1.032;1.118] |  | 1.011 | [0.999;1.024] |  | 1.109 | [0.811;1.518] |  | 1.081 | [1.071;1.092] |
| Q4 (Oct.-Dec.) | 1.065 | [1.024;1.108] |  | 1.017 | [1.005;1.029] |  | 0.894 | [0.645;1.240] |  | 1.038 | [1.028;1.048] |
| Outcome-specific additional covariables | |  |  |  |  |  |  | |  |  | |
| Diabetes mellitus | 0.744 | [0.720;0.769] |  | 1.034 | [1.023;1.045] |  | 1.001 | [0.772;1.298] |  | *NA* | |
| CVD | 1.835 | [1.766;1.907] |  | 1.409 | [1.393;1.424] |  | *NA* | |  | *NA* | |
| Cardiac surgery | 0.995 | [0.883;1.121] |  |  | *NA* |  | *NA* | |  | *NA* | |
| COPD |  | *NA* |  | 1.147 | [1.133;1.162] |  | *NA* | |  | *NA* | |
| Renal diseases |  | *NA* |  | 1.135 | [1.121;1.148] |  | 1.006 | [0.757;1.339] |  | *NA* | |
| Liver diseases |  | *NA* |  | 1.135 | [1.121;1.148] |  | 2.291 | [1.775;2.958] |  | *NA* | |
| Approximate significance of smooth terms: | | |  |  |  |  |  | |  |  | |
|  | edf | p-value |  | edf | p-value |  | edf | p-value |  | edf | p-value |
| Follow-up | 2.938 | <0.001 |  | 2.767 | <0.001 |  | 2.957 | <0.001 |  | 2.999 | <0.001 |
| Age in years | 5.994 | <0.001 |  | 4.973 | <0.001 |  | 4.825 | <0.001 |  | 8.390 | <0.001 |
| Adjusted hazard ratio (aHR) \| 95% confidence interval (CI) [lower confidence level; upper confidence level] \| reference (ref.) \| Charlson comorbidity index (CCI) \| Quarter 1-4 (Q1-4): January-March, April-June, July-September, October-December \| cardiovascular disease (CVD) \| chronic lower respiratory diseases (COPD) \| not applicable (NA) \| estimated degrees of freedom (edf). | | | | | | | | | | | |

Table S6. Results from PAMM regression models in sensitivity analyses, cohort 1-4

| **Analysis** | **Cohort 1: aortic aneurysm/dissection** | | **Cohort 2: cardiac arrhythmia/ sudden cardiac death** | | **Cohort 3: acute toxic liver injury/ acute liver failure** | | **Cohort 4: all-cause mortality** | |
| --- | --- | --- | --- | --- | --- | --- | --- | --- |
|  | **aHR** | **[95% CI]** | **aHR** | **[95% CI]** | **aHR** | **[95% CI]** | **aHR** | **[95% CI]** |
| Healthiest* only (365-day risk window) | | *NA* |  | *NA* |  | *NA* | 1.320 | [1.231;1.414] |
| Healthiest* only (92-day risk window) |  | *NA* |  | *NA* |  | *NA* | 1.351 | [1.192;1.532] |
| Per-protocol censoring | 1.103 | [1.078;1.128] | 1.079 | [1.071;1.087] | 1.356 | [1.313;1.399] | 1.255 | [1.247;1.262] |
| Excluding hospitalisation at baseline | 1.085 | [1.054;1.118] | 1.050 | [1.041;1.060] | 1.301 | [1.241;1.363] | 1.172 | [1.160;1.183] |
| Hospitalisation during follow-up: |  |  |  |  |  |  |  |  |
| 0 days | 1.080 | [1.046;1.114] | 1.049 | [1.039;1.059] | 1.191 | [1.126;1.260] | 1.094 | [1.088;1.099] |
| 1-7 days | 1.025 | [0.977;1.075] | 0.999 | [0.985;1.014] | 1.351 | [1.272;1.435] | 1.095 | [1.086;1.104] |
| ≥ 8 days | 1.037 | [0.981;1.098] | 1.014 | [0.994;1.034] | 1.269 | [1.202;1.340] | 1.080 | [1.075;1.086] |
| Defined daily dose: |  |  |  |  |  |  |  |  |
| Low | 1.021 | [0.939;1.110] | 1.026 | [1.001;1.052] | 1.120 | [0.993;1.264] | 1.107 | [1.086;1.129] |
| Medium | 1.065 | [1.034;1.098] | 1.030 | [1.020;1.039] | 1.270 | [1.218;1.324] | 1.197 | [1.188;1.207] |
| High | 1.086 | [1.038;1.137] | 1.094 | [1.078;1.111] | 1.593 | [1.500;1.693] | 1.427 | [1.409;1.445] |
| Adjusted hazard ratio for fluoroquinolone-episode (aHR) \| 95% confidence interval (CI) [lower confidence level; upper confidence level] \| *restricting the all-cause mortality cohort to individuals without CCI, drugs dispensed, and hospitalised days at baseline as a proxy for not being able to adjust for comorbidities \| not applicable (NA). | | | | | | | | |
